# Exploring the relationship between lifestyles, diets and genetic adaptations in humans

**DOI:** 10.1186/s12863-015-0212-1

**Published:** 2015-05-28

**Authors:** Cristina Valente, Luis Alvarez, Sarah J. Marks, Ana M. Lopez-Parra, Walther Parson, Ockie Oosthuizen, Erica Oosthuizen, António Amorim, Cristian Capelli, Eduardo Arroyo-Pardo, Leonor Gusmão, Maria J. Prata

**Affiliations:** IPATIMUP, Institute of Molecular Pathology and Immunology of the University of Porto, Porto, Portugal; Faculty of Sciences, University of Porto, Porto, Portugal; Department of Zoology, University of Oxford, Oxford, UK; Departamento de Toxicología y Legislación Sanitaria, Facultad de Medicina, Universidad Complutense de Madrid, Madrid, Spain; Institute of Legal Medicine, Innsbruck Medical University, Innsbruck, Austria; Penn State Eberly College of Science, University Park, Pennsylvania, USA; School of Medicine, University of Namibia, Windhoek, Namibia; DNA Diagnostic Laboratory (LDD), State University of Rio de Janeiro (UERJ), Rio de Janeiro, Brazil

**Keywords:** Diet adaptations, Signals of natural selection, Africa Sub-Saharan

## Abstract

**Background:**

One of the most important dietary shifts underwent by human populations began to occur in the Neolithic, during which new modes of subsistence emerged and new nutrients were introduced in diets. This change might have worked as a selective pressure over the metabolic pathways involved in the breakdown of substances extracted from food. Here we applied a candidate gene approach to investigate whether in populations with different modes of subsistence, diet-related genetic adaptations could be identified in the genes *AGXT*, *PLRP2*, *MTRR*, *NAT2* and *CYP3A5.*

**Results:**

At *CYP3A5,* strong signatures of positive selection were detected, though not connected to any dietary variable, but instead to an environmental factor associated with the Tropic of Cancer. Suggestive signals of adaptions that could indeed be connected with differences in dietary habits of populations were only found for *PLRP2* and *NAT2*. Contrarily, the demographic history of human populations seemed enough to explain patterns of diversity at *AGXT* and *MTRR,* once both conformed the evolutionary expectations under selective neutrality.

**Conclusions:**

Accumulated evidence indicates that *CYP3A5* has been under adaptive evolution during the history of human populations. *PLRP2* and *NAT2* also appear to have been modelled by some selective constrains, although clear support for that did not resist to a genome wide perspective. It is still necessary to clarify which were the biological mechanisms and the environmental factors involved as well as their interactions, to understand the nature and strength of the selective pressures that contributed to shape current patterns of genetic diversity at those *loci*.

**Electronic supplementary material:**

The online version of this article (doi:10.1186/s12863-015-0212-1) contains supplementary material, which is available to authorized users.

## Background

The most remarkable dietary change over the recent history of human populations was that associated with the change from food collection to food production [1], which occurred independently and in different times in separate parts of the world marking the beginning of the Neolithic, a transition that in some regions dates back to 12,000 years ago. The domestication of plants and animals prompted the conditions that would brought about new modes of subsistence as well as new food habits as a consequence of the shift in the availability and exploitation of dietary resources [1, 2]. Genetic adaptations to dietary specializations are thought to have represented advantageous evolutionary solutions in humans, however it is still unclear the extent to which dietary factors have created selective pressures acting on genes that play roles in food-related metabolic pathways. Recent studies have revealed genomic signatures of adaptations likely driven by diet-related pressures [1, 3, 4]. In addition, candidate genes approaches had already provided tight evidence for genetic adaptations to differences in nutrient consumption such as at the lactase and amylase genes [5-10].

Other metabolic-related genes have been hypothesized to constitute dietary adaptations, among which are included: *AGXT,* coding for alanine:glyoxylate aminotransferase, the enzyme responsible for the transamination of glyoxylate into glycine [11-13]; *PLRP2,* coding for pancreatic lipase-related protein 2, involved in galactolipids hydrolysis, [14-17]; *MTRR,* encoding for methionine synthase reductase, an enzyme acting in the complex folate pathway [15, 18]; *NAT2* coding for *N*-acetyltransferase 2, a phase-II enzyme involved in the detoxification of a wide number of xenobiotics [15, 19-23]; and *CYP3A5,* coding for cytochrome P-450 3A5, a member of the CYP3A enzymes that are involved in the oxidative metabolism of many endogenous substrates and xenobiotics, which is implied in sodium homeostasis [24-27].

Genetic variation in *AGXT* was tentatively linked with meat content in diets, *PLRP2* with richness in cereals [15], both *MTRR* and *NAT2* with availability of folate in foods and *CYP3A5* with health conditions that are influenced by dietary salt intake [24, 27]. However, for these 5 genes results so far obtained were either contradictory (e.g. *AGXT*), or not yet replicated (e.g. *MTRR* and *PLRP2*), or not clear enough to ascertain whether they can indeed represent genetic adaptations to any dietary variable. This prompted us to address the issue applying of genetic adaptation within those genes.

Thus, assuming that current modes of subsistence are still good surrogates of main diets in which populations have traditionally relied, the aim of this study was to gain further insights into the relationship between diet-related variables in populations and patterns of diversity at variations in above mentioned five genes.

Functional variants within *AGXT*, *PLRP2, MTRR, NAT2* and *CYP3A5* were examined in six sub-Saharan populations with distinct modes of subsistence and also in one European population that was also screened to generate a non-African reference group. Results were then combined with previously published information for other African and Eurasian populations to evaluate the contribution of geography and mode of subsistence or other diet-related variables to explain the patterns of genetic diversity observed for the five genes.

## Results

### Locus by locus analysis

The observed genotypic distributions (Additional file 1: Table S1) did not revealed significant departures from Hardy-Weinberg expectations after applied the Bonferroni’s correction for multiple tests. Estimates of allele frequencies for the five *loci* in the seven studied populations are shown in Table 1 and for each locus results here and previously obtained will be dissected in the following sections.

**Table 1:** Derived allele frequencies

POPULATION	c.32C > T *(AGXT*)	c.1074G > A (*PLRP2*)	c.1130A > G (*MTRR*)	c.191G > A (NAT2*14)	c.341 T > C (NAT2*5)	c.590G > A (NAT2*6)	c.857G > A (NAT2*7)	c.219-237G > A (*CYP3A5*)
ANG	0.0000 ± 0.0000	0.3261 ± 0.0691	0.5294 ± 0.0856	0.1522 ± 0.0530	0.2046 ± 0.0748	0.3636 ± 0.0725	0.0000 ± 0.0000	0.2400 ± 0.0604
EQG	0.0482 ± 0.0166	0.3214 ± 0.0360	0.3563 ± 0.0363	0.0977 ± 0.0225	0.3588 ± 0.0536	0.1786 ± 0.0296	0.0233 ± 0.0115	0.1429 ± 0.0270
MOZ	0.0370 ± 0.0257	0.2333 ± 0.0546	0.5500 ± 0.0642	0.1429 ± 0.0540	0.2500 ± 0.0884	0.2857 ± 0.0697	0.0000 ± 0.0000	0.1167 ± 0.0414
UGN	0.0727 ± 0.01751	0.3945 ± 0.0331	0.3835 ± 0.0339	0.0699 ± 0.0187	0.3902 ± 0.0575	0.3085 ± 0.0337	0.0055 ± 0.0055	0.2336 ± 0.0289
BPY	0.0147 ± 0.0146	0.18912 ± 0.0455	0.3846 ± 0.0551	0.0263 ± 0.0184	0.1842 ± 0.0536	0.2568 ± 0.0508	0.0000 ± 0.0000	0.1447 ± 0.0404
KNA	0.0000 ± 0.0000	0.0242 ± 0.0138	0.1371 ± 0.0309	0.0000 ± 0.0000	0.0656 ± 0.0239	0.0484 ± 0.0193	0.0968 ± 0.0266	0.2097 ± 0.0366
PTG	0.1915 ± 0.0406	0.5106 ± 0.0516	0.1383 ± 0.0356	0.0000 ± 0.0000	0.5000 ± 0.0903	0.2021 ± 0.0414	0.0532 ± 0.0232	0.9022 ± 0.03010

Populations’ abbreviations as referred in material and methods section

### AGXT

In the *AGXT* gene, we studied the variant c.32C > T, concerning which the derived allele T had been previously suggested to play an adaptive role in populations traditionally relying in meat-rich diets [11, 28]. The hypothesis was specifically investigated by Caldwell et al. [11] who reported on frequency data sustaining the model, a conclusion for which much accounted the observation of the highest frequency of the derived allele in the Sweden Saami, who have a long history of consuming high amounts of animal products [11, 28]. Though, later, revisiting the question with a better coverage of Central Asian populations Ségurel et al. [13] failed to find increased allele frequencies across populations with diets richer in meat comparatively to those less meat rich, challenging this way the adaptive model proposed for the variation.

In this study, in terms of meat content in diets of African populations, we have assumed that in general farmers rely less in meat than pastoralists or hunter-gatherers, in accordance with a recent review from ethnographic compilations of hunter-gatherer diets indicating that animal food comprises their dominant energy source [29]. Among the 6 sub-Saharan populations examined, the frequency of the derived allele at c.32C > T ranged from 0 to 7.27 % without showing any pattern of variation that could be connected with mode of subsistence or meat content in diets of populations. For instance, it was absent both from the farmers from Angola and from the hunter-gatherers Khoisan, although the first are representative of less meat consumers groups while the second are from more meat consumers ones. In the sample from Portugal, considered to be a farming population with a mixed diet reasonably balanced regarding animal and plant food resources, the derived allele reached 19.15 %, a frequency higher than registered in any of the African populations regardless of its mode of subsistence or reliance upon meat.

To integrate our results in a more comprehensive distribution, data for c.32C > T was retrieved from the literature on populations for which information on the relative predominance of meat in their diets was available (Additional file 2: Table S2). There were results only for populations from Africa and Eurasia, among which the average frequency of the derived allele was 0.081 across the set of populations assigned to have high meat consumption, while it was, 0.133, across the populations with low-meat consumption. Actually neither the overall differences in allele frequencies within the “low-meat” and “high-meat” groups were statistically significant (*P* = 0.0710, One-Way ANOVA), nor the trend in the frequency distribution sustained the hypothesis that the allele could be positively selected in meat-rich diet populations.

Furthermore, if the broad geographical distribution of c.32C > T in Africa and Eurasia conformed well the major population clusters commonly identified by random neutral genetic markers, intriguingly in Asia, where there is a high dispersion of gene frequencies, the extreme values were reported for two populations in rather close geographical proximity but with distinct traditional lifestyles: in the Tajiks, a group of sedentary agriculturalists from Western Tajikistan the derived allele was very well represented (26.9 %), whereas in the Kazaks from Western Uzbekistan, who are traditionally nomadic herders whose diet mainly consists of meat, milk and dairy products, the allele only occurred marginally (1.7 %).

From these analyses, no connection emerged between the frequency distribution of c.32C > T in *AGXT* and lifestyle of populations.

### PLRP2

In this gene we focused on c.1074G > A, a variant that causes a premature truncation of the pancreatic lipase-related protein 2 resulting in a more active version of the enzyme. In a very recent genome-wide scan for selection in human populations, Hancock et al. [15] identified in this variant a convincing signal of adaptation to a dietary specialization, since the derived allele was found to be significantly more common in populations relying in diets with high content in cereals (farmers) than in other populations.

As long as we know, the association was not further investigated except in the present study, where among the screened African groups, the derived allele was detected to be quite common in the three farmers’ groups (23.3 % - 32.6 %) as well as in the herders from Uganda (39.5 %). Comparatively, the two hunter-gatherers groups showed lower frequencies, specially the Ju/hoansi (2.42 %). The sample from Portugal showed the highest frequency in this study with, 51.1 % (Table 1).

**Fig. 1:**
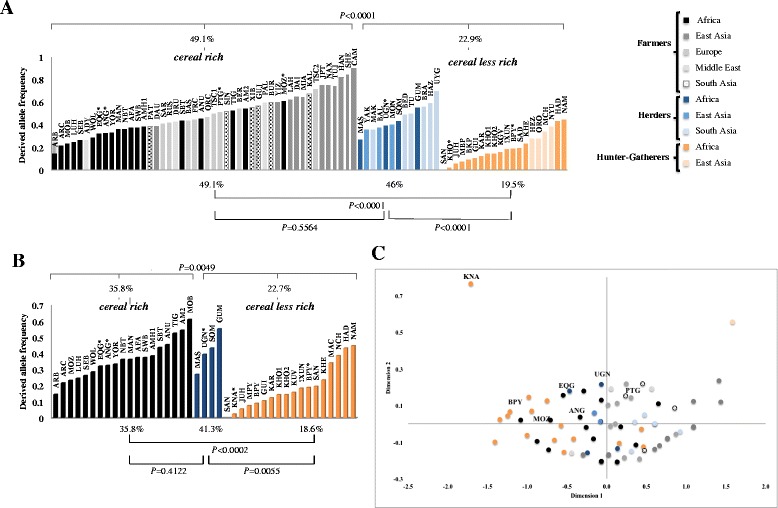
Allele frequencies and MDS plot for *PLRP2. P* values of ANOVA One-Way test in **(A)** African + Eurasian and **(B)** African populations’ group; MDS plot of pairwise genetic distances between populations **(C)**. In the MDS plot different colors represent distinct lifestyles: hunter-gatherers (*orange*), herders (*blue*) and farmers populations (*black*). *populations addressed in this study. Populations’ abbreviations are referred in Material and Methods section

As a whole, our results do not conflict with the hypothesis that the distribution of c.1074G > A might be related to the weight of cereals in diets, in the sense that at least within Africa, farmers populations tended to have higher frequencies of the derived allele compared to hunters-gatherers who rely less in cereals. These results were then put in a wide-ranging context, recruiting information on c.1074G > A for African and Eurasian populations from several sources, and maintaining the classification in populations that specialize and that do not specialize on cereals when originally presented (Additional file 2: Table S2). As shown in Fig. 1A and B, the frequency of the truncated allele was found to be more common across populations with cereal-rich diets (average frequency 35.8 % in Africa; 49.1 % in Eurasia + Africa) than across those less dependent on cereals (average frequency 22.7 % in Africa; 22.9 % in Eurasia + Africa), differences that were statistically significant either in Africa (*P* = 0.0050, One-Way ANOVA) or in Eurasia + Africa (*P* = <0.0001, One-Way ANOVA). Comparing herders and hunter-gatherers, both integrated in the group of cereal less rich populations (Fig. 1), mean frequency was respectively 41.3 % and 18.6 % in Africa, and 46 % and 19.5 % in Eurasia + Africa, with both differences being again statistically significant (*P =* 0.0060 for Africa; *P* = <0.0001, for Eurasia + Africa, One-Way ANOVA). Considering Africa and Eurasia together, the trend that can be extracted from the whole data points to a decreasing frequency gradient of the derived allele at c.1074G > A from populations more specialized on cereals towards those less relying on them, as was also captured by the MDS plot shown in Fig. 1C, where it is visible some structure between hunter-gatherer, herder and farmer populations.

As a whole, these results suggest that diversity at *PLRP2* was shaped by selective pressures that differed according to populations’ lifestyle.

### MTRR

Within *MTRR* we examined the common variation affecting levels of enzymatic activity c.1130A > G, since it was another candidate adaptive genetic variation identified in the before mentioned genome-wide study [15]. Before, *MTRR* had received high attention in association studies, having been implicated, for instance, with risk for *spina bifida* [18]. However, its adaptive role to dietary specializations was addressed in only one work where c.1130A > G was found to be strongly correlated with diets containing mainly the folate-poor foods roots and tubers [15]. The results obtained in this work revealed that the derived allele was quite common in most African groups, peaking in the agriculturists from Angola and Mozambique with values of 0.529 and 0.550, respectively (Table 1). Both estimates are similar to that described in the Yoruba (0.548) the only African group with a diet principally relying on roots and tubers addressed in a previous study [15]. So, at least in Africa high frequencies of this allele can be found in populations without having such a dietary specialization. Furthermore, no indication arose that the distribution of c.1130A > G could be correlated to the dietary availability in folates, which is generally thought to be lower in non-forager populations (agricultural and pastoral) than in hunter-gatherers [22]. In fact, in the hunter-gatherers Baka, in the herders from Uganda and in the farmers from Equatorial Guinea, the derived allele occurred at similar frequencies (0.385, 0.384, 0.356, respectively) despite the differences in mode of food production. In the hunter-gatherers Ju/honasi from Namibia, the allele occurred at the lowest frequency in Africa (0.137) but with a magnitude similar to that found in the European sample (0.138), considered as a representative of an agriculturalist society (Table 1). To interpret our results under a wide framework of African and Eurasian populations, frequency data were recruited once more from the literature (Additional file 2: Table S2), and the combined information allowed to realize that the distribution of c.1130A > G fitted well the pattern generally provided by neutral markers, not appearing to be influenced by the mode of subsistence or the relative folate content in diets of populations from Eurasia and Africa. In East Asia, for instance, the two highest values of the derive allele were present in the Tu (0.4), nomadic herders, and in the Hezhen (0.333), mainly hunters and fishers, but nonetheless in the foragers Orogen and Yakut, who also live in East Asia, the allele was absent or very rare (Additional file 2: Table S2).

So, for the variation c.1130A > G in *MTRR,* the current patterns of diversity do not indicates that it could represent an adaptation to the mode of subsistence of human populations.

### NAT2

The dietary availability in folates had also been previously hypothesized to be a modulator of genetic diversity at the gene that encodes for *NAT2* (*N*-acetyltransferase 2) [22]. Individuals can be classified in fast, intermediate or slow acetylator phenotypes, which are determined by the haplotypic composition defined by genetic variations at the *NAT2* locus. Evidence for the diet-related hypothesis provided by Luca et al. [22] was reinforced with the recent findings by the same people [1], based on a more comprehensive analysis of *NAT2* worldwide genetic diversity, that were also compatible with a model holding that the slow acetylator phenotypes were selectively favored in populations relying in dietary regimens with reduced folate supply, whereas the fast acetylators were neutral or even advantageous in the presence of folate-rich diets, as those thought to be fulfilled by hunter-gatherers. To extent the population coverage of previous works, frequencies of *NAT2* haplotypes and acetylator phenotypes were also estimated in this study (Additional file 3: Table S3). The distribution of haplotypes was very heterogeneous across African populations, but in line with previous observations the prevalence of the slow acetylator phenotype in the two hunters-gatherers groups (Khoisan, 1.6 %; Baka Pygmies, 13.5 %) was significantly much lower than in the three agriculturalists groups or in the Ugandan pastoralists, all displaying values up to 37.4 % (*P* = 0.0139, One-Way ANOVA). In the Portuguese the slow acetylator phenotype accounted for the high proportion of 52.2 %, which falls within the range typical from other European populations [21].

Next, we contrasted our data with other results before published for Eurasian and African populations (Additional file 2: Table S2), confining the analysis to c.590G > A, which defines allele NAT2*6, because it was the variation with more information accumulated for populations representatives of the three modes of subsistence.

**Fig. 2:**
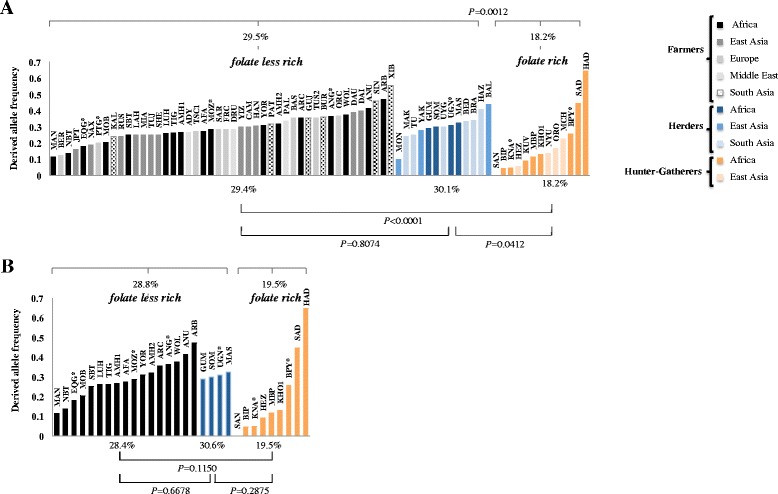
Allele frequencies for *NAT2. P* values of ANOVA One-Way test in **(A)** worldwide and **(B)** African populations’ group hunter-gatherers (*orange*), herders (*blue*) and farmers populations (*black*), *populations addressed in this study. Populations’ abbreviations are referred in material and methods section

From Fig. 2A, which shows the allelic distribution of c.590G > A across Africa and Eurasia, it becomes clear that its prevalence is scarcely influenced by the continent where populations are located. However, some connection arises with systems of food production and acquisition given that in the whole set of African and Eurasian populations foraging groups tended to exhibit statistically significant lower frequencies of the derived allele compared to populations dependent on agricultural and pastoral resources (see in Fig. 2B the *P*-values of One-Way ANOVA). Between pastoralists and agriculturalists, no significant differences were detected, which means that the clustering of c.590G > A frequencies only showed correspondence with populations that are food producers or food collectors, an observation that otherwise fully meets that reported by Sabbagh et al. [21], and the results even more recent published by the same team [30].

In brief, our analyses reinforce previous indications that *NAT2* has evolved under a selective factor influenced by human diet.

### CYP3A5

With regard to *CYP3A5,* we screened the intronic variation c.219-237G > A, commonly referred to CYP3A5*1/*3 polymorphism, in which the derived allele A results in a premature stop codon that reduces protein expression. It has been firmly demonstrated that the variation possesses a very unusual worldwide distribution whereby the frequency of CYP3A5*3 is significantly correlated with latitude [24].

CYP3A5*1/*3 likely influences salt and water retention and risk for salt-sensitive hypertension [24], exerting an effect on blood pressure that is determined by interactions with dietary salt intake [27,31]. Since anthropological evidence indicates that diet of hunting and gathering people is usually characterized by low level of salt intake, being often considered as a surrogate of the preagricultural humans’ diet, lately praised as a model of well balanced food consumption [32], we asked whether diversity at CYP3A5*1/*3 could be related with diet of populations.

**Fig. 3:**
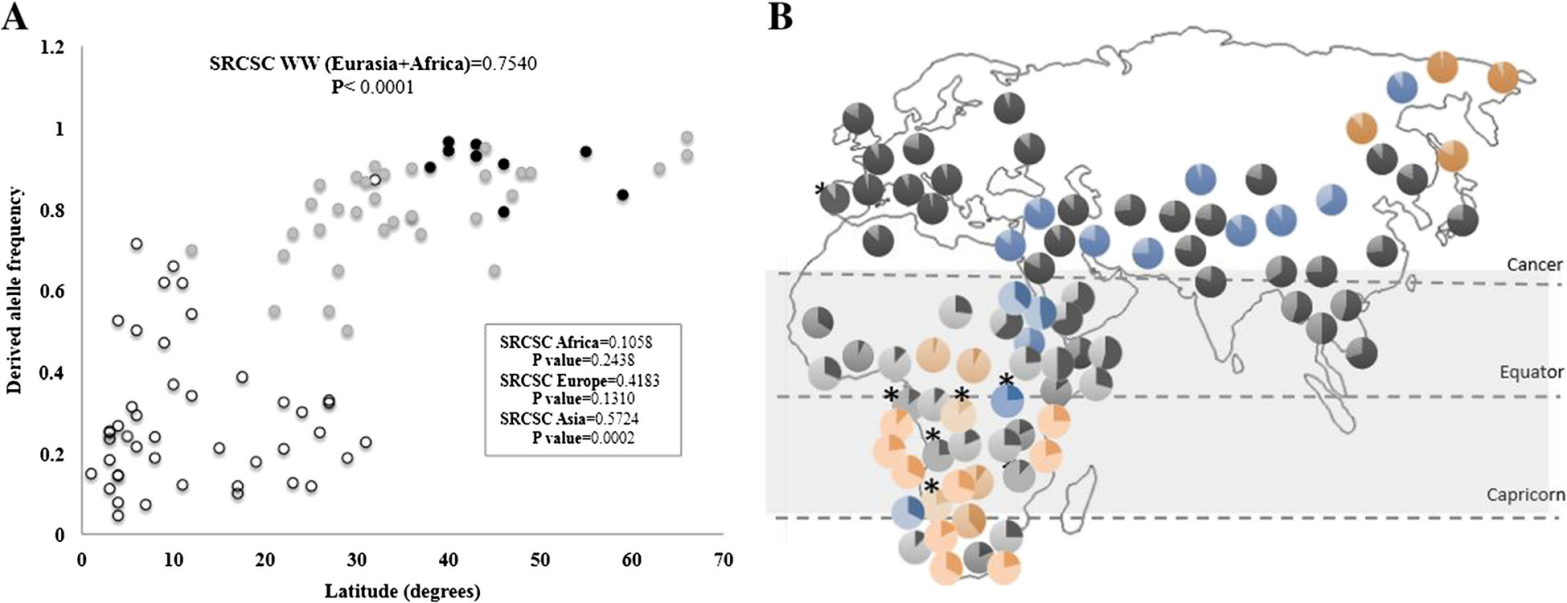
Distribution of CYP3A5*3 in Africa and Eurasia and correlation with latitude. Correlation plot between latitude and allele frequencies in African (*open dots*), European (*black dots*) and Asian populations (*grey dots*) **(A)**. Map representing the distribution of CYP3A5*3 across Africa and Eurasia **(B)**. ancestral allele frequency (*light pie*) and the derived allele (*dark pie*); hunter-gatherers (*orange pie*), herders (*blue pie*) and farmers populations (*black pie*), *populations addressed in this study

Thus, we screened the variation in the six African populations, among whom the derived allele was only moderately represented, but suggestively it was in two farmer groups that the lowest and the highest frequencies were found (11.7 % and 24.0 % in the groups from Mozambique and Angola, respectively), disfavoring thus any link between lifestyle and differences in allele frequency across populations. In the Portuguese, the allele reached the very elevated value of 90.2 %, which it is usual in populations from Europe where CYP3A5*3 varies quiet narrowly being near-fixation in most populations [24]. Again, our data were combined with those retrieved from the literature (Additional file 2: Table S2), and with an enlarged coverage of African and Eurasian populations, we confirmed in fact that the frequency of the low expressor allele significantly increased with distance from the equator (Fig. 3A) (SRCSC = 0.7540; *P* < 0.0001). When the relationship was assessed separately in each of the three continents, no significant rank correlation was observed in Africa (SRCSC = 0.1058; *P* = 0.2438) or in Europe (SRCSC = 0.4183; *P* = 0.1310), but in Asia the correlation coefficient was again statistically significant (SRCSC = 0.5724; *P* < 0.0002). Interestingly, in Asia, where the average allele frequency was 0.793, the significant correlation can be explained since the lowest values are consistently present in populations from the South of the continent, located very near or already inside the intertropical zone. In Africa, the frequency of the allele drastically declines to an average value of 0.286 when inferred from a panel of populations’ majority located inside the tropical zone. In Europe, which is fully situated in a temperate climatic region, the average frequency reaches 0.903. Therefore, being or not located in the tropical zone seems to be a factor that strongly influences the distribution of CYP3A5*1/*3 alleles (see Fig. 3B).

These analyses led to conclude that *CYP3A5* was the target of a selective factor determined by the geographic location of human populations.

### Hierarchical AMOVA

Hierarchical AMOVA was performed to determine the relative contribution of geography, mode of subsistence and different diet-related variables to the genetic structure observed in the SNPs at *AGXT*, *PLRP2, MTRR*, *NAT2* (only for that defining NAT2*6) and *CYP3A5*, hereinafter referred for simplicity as uniquely by their gene symbols (Table 2).

**Table 2:** AMOVA analysis under different criteria

	c.32C > T (*AGXT*)	*P*-value	c.1074G > A (*PLRP2*)	*P*-value	c.1130A > G (*MTRR*)	*P*-value	c.590G > A (*NAT2*)	*P*-value	c.219-237G > A (*CYP3A5*)	*P*-value
*Mode of subsistence*	**1.5**	**0.0355**	**8.8**	**0.0000**	−0.6	0.8793	**1.6**	**0.0046**	4.9	0.0542
*Main diet component*	0.4	0.1478	**6.5**	**0.0001**	−0.2	0.4712	**3.0**	**0.0019**	2.5	0.1004
*Geography*	**3.9**	**0.0001**	**8.5**	**0.0000**	**3.4**	**0.0003**	0.2	0.2149	**40.4**	0.0000
*Latitude*	**-**	**-**	**-**	**-**	**-**	**-**	**-**	**-**	**37.8**	**0.0000**
*Above/below Tropic of Cancer*	**-**	**-**	**-**	**-**	**-**	**-**	**-**	**-**	**44.9**	**0.0000**

Significant differences are highlighted in **bold**

Geography was found to significantly account to explain the total genetic variance across Africa and Eurasia at *AGXT, PLPR2, MTRR,* and *CYP3A5*, but not at *NAT2*. The contribution of geography was especially high in *CYP3A5* in which it amounted to a very high proportion, 40.4 % of total diversity. For this variation it was further assessed the effect of i) latitude and ii) the location North and South the Tropic of Cancer, leading to realize that for *CYP3A5* the highest value of *F*_CT_ (which measures the proportion of variance among groups) was achieved when populations North of the Tropic of Cancer were grouped against the southern ones, attaining then 44.9 % of total diversity.

Concerning mode of subsistence, it was found to be a considerable modulator of diversity at *PLRP2*, explaining 8.8 % of the total diversity at the locus, while also accounting to residual proportions of diversity at *NAT2* (1.6 %) and *AGXT* (1.5 %). When the criterion to group populations was the content in diets of cereals (for *PLPR2*), meat (for *AGXT*), folates (for *MTRR* and *NAT2*) or salt (for *CYP3A5*), significant *F*_CT_ values were only observed at *PLRP2,* in which the more or less reliance in cereals contributed to 6.5 % of the total variance, and at *NAT2,* where differences in the dietary richness in folates explained 3 % of the locus diversity.

### Signals of selection

To dissect better whether from the levels of genetic differentiation across Africa and Eurasia signs of selection could be captured, we used a conventional *F*_ST_-based approach that assumes that genetic differentiation among populations is expectedly higher or lower for *loci* under directional or balanced selection, respectively, expected under neutrality.

Viewing that, we have firstly generated null sampling distribution of the empirical *F*_ST_ employing two different models, the finite Island Model (IM), which assumes the classical island model at migration-drift equilibrium [33]; and the Hierarchical Island Model (HIM), in which populations samples are assigned to different groups, allowing for increased migration rates between populations within groups than between groups [34]. Besides portraying more realistically the demographic history of human populations, HIM was shown to produce a low rate of false positive signs comparatively to IM, when used to test *loci* for selection [34].

**Fig. 4:**
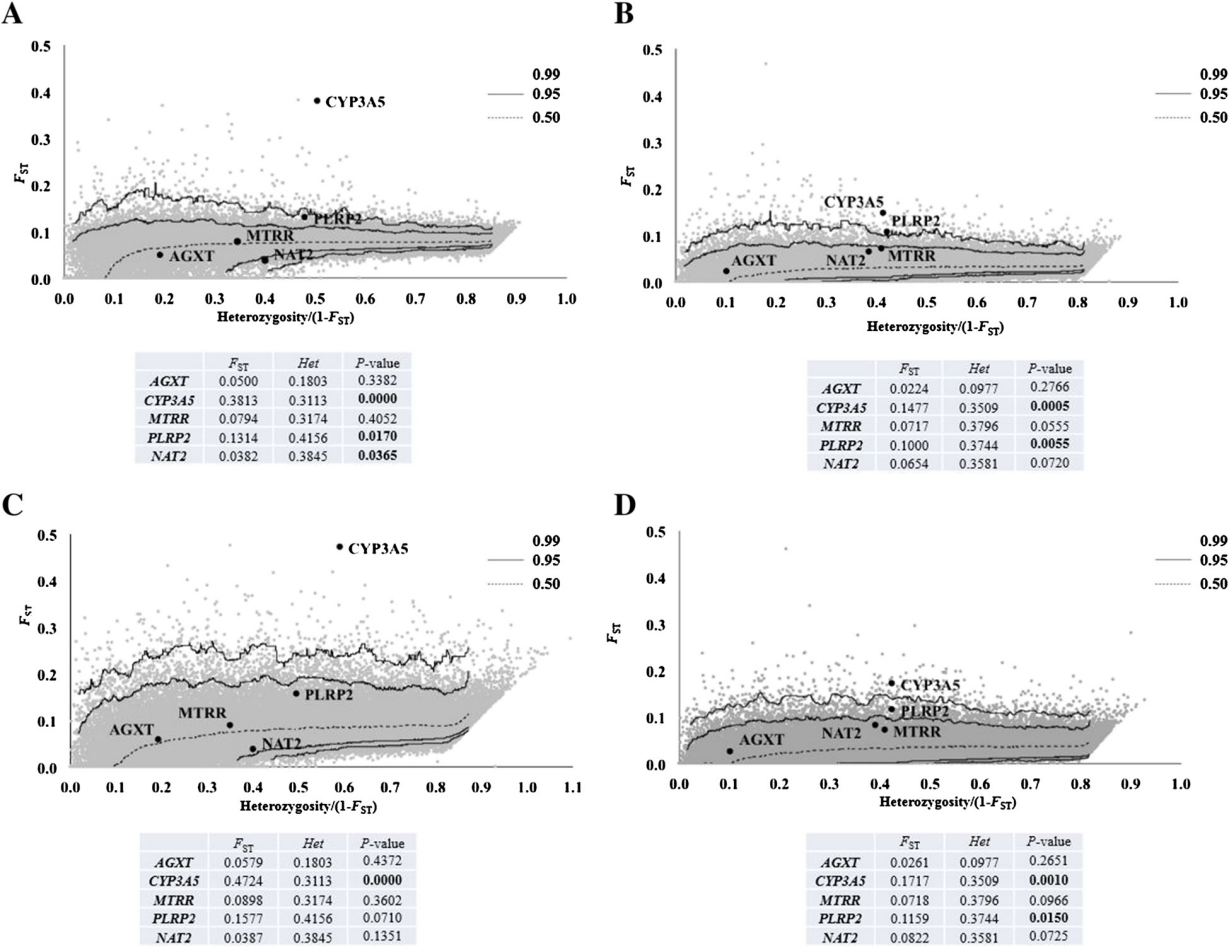
Joint distribution of *F*_ST_ vs. Scaled Heterozygosity expected under two neutral models. Joint distributions in African + Eurasian (**A**) and African populations (**B**) under Island Model (IM); and joint distributions in African + Eurasian (**C**) and African populations (**D**) under Hierarquical Island Model (HIM). It is represented the 99 % confidence regions of the null distribution. Black dots represent the observed measures in the studied genes, referred for simplicity as uniquely by their gene symbols; significant differences after Bonferroni’s correction for multiple tests are highlighted in bold

The simulated null-distributions are presented in Fig. 4 where are also shown the *F*_ST_ values plotted against scaled heterozygosity estimated for the SNPs at *AGXT*, *PLRP2, MTRR, CYP3A5* and *NAT2* [35].

Considering simultaneously Africa and Eurasia and using as reference the IM distribution, the *F*_ST_s for *MTRR* and *AGXT* did not differed significantly from the null expectations (Fig. 4A). By contrast, the global differentiations at *PLRP2, NAT2* and *CYP3A5*, all lied outside the 95 % confidence region of the neutral distribution, though showing departures with opposite directions: whereas the *F*_ST_ coefficient for *NAT2* was significantly smaller than expected, the coefficients for *CYP3A5* and *PLRP2* were both significantly larger (*P*-values in Fig. 4A). The outlier position is especially remarkable in the case of *CYP3A5* that presented the exceedingly high *F*_ST_ coefficient of 0.3813, almost five times greater compared to the average empirical neutral level of 0.079 between African and Eurasian populations. These results suggest that *NAT2* could have been under balanced or negative selection whist both *PLRP2* and *CYP3A5* might well have been modeled by positive selection. Taken into account the *F*_ST_ null distribution simulated under the HIM (Fig. 4C), the *F*_ST_s for *NAT2* and *PLRP2* lost the condition of significant outliers and the unique differentiation that remained significantly higher than the neutral expectations was at *CYP3A5.* Simulations were also carried out considering separately Africa and Eurasia. While in Eurasia none of the five assessed SNPs revealed to be outsiders in the distributions simulated under the simple or the hierarchical island models (results not shown), noteworthy in Africa the differentiations at *PLRP* and *CYP3A5* were significantly higher than expected under the neutral expectations derived from the two demographical models (Fig. 4B and D).

### LD patterns

In order to assess whether the examined genetic variants were in fact those responsible for the selective signals detected *PLRP2, NAT2* and *CYP3A5,* we explored the patterns of *linkage disequilibrium* (LD) surrounding each of the three genes, viewing which a genomic window was considered that encompassed the adjacent genes. In Table 3 are presented the non-synonymous variants showing significant *D’* and *r*^*2*^ values with our target SNPs, identified in African populations, which were the unique with genome data available. The correspondent LD plots for each gene across different African populations are present in supplementary material (Additional file 4: Figure S1, Additional file 5: Figure S2, Additional file 6: Figure S3, Additional file 7: Figure S4, Additional file 8: Figure S5, Additional file 9: Figure S6, Additional file 10: Figure S7). For *CYP3A5* and *NAT2* no significant LD was detected with neighbor genes. Within each of the two genes, high LD was only found between our target SNP at *NAT2* and the linked variants rs1801280 and rs1208, both associated with decreased enzyme activity like rs1799930. Although this makes it difficult to discriminate the effects of the three variants, we can conclude that the selective signal detected at *NAT2* is related with variations that affect enzyme activity in a similar direction. As for the gene *PLRP2*, it was found to be located in a region of considerable LD with *PLRP1*, a downstream gene that codes for pancreatic lipase-related protein 1. Within *PLRP1* two non-synonymous (rs2305204 and rs1049125), whose functional consequences are unknown, are in strong LD with our target SNP at *PLRP2*, which in addition was at high LD with rs475199, a non-synonymous substitution of unknown functional effect, also located in *PLRP2*.

**Table 3:** Linkage Disequilibrium including *D’* and *r* ^*2*^ parameters

Gene (target SNP)	Non-synonymous SNP	*D’*	*r* ^*2*^	Gene location	DNA changes	Functional consequences	Reference
*PLRP2* (rs4451995)	rs2305204	1.0	0.036	*PLRP1*	c.1242G > C	-	[58]
		n.d.	n.d.				[57]
		n.d.	n.d.				[59]
	rs1049125	0.884	0.052	*PLRP1*	c.1382 T > C	-	[58]
		n.d.	n.d.				[57]
		n.d.	n.d.				[59]
	rs4751996	0.981	0.962	*PLRP2*	c.1084G > A	-	[58]
		n.d.	n.d.				[57]
		n.d.	n.d.				[59]
*NAT2* (rs1799930)	rs1801280	n.d.	n.d.	*NAT2*	c.341 T > C	Associated with slow acetylator due to N-acetyltransferase enzyme variant (acetylation slow phenotype)	[58]
		1.0	0.142				[57]
		n.d.	n.d.				[59]
	rs1208	n.d.	n.d.	*NAT2*	c.803G > A		[58]
		1.0	0.360				[57]
		n.d.	n.d.				[59]

n.d. no data available

## Discussion

The analysis of patterns of human genetic diversity at wide geographical scales can disclose remarkable features difficultly explained by demographic events or pure neutral processes, that rather might represent the first symptoms of environmental adaptations.

In this study, we draw attention to variations in *AGXT*, *PLRP2*, *MTRR*, *NAT2* and *CYP3A5*, five genes assumedly involved in the metabolism of substances (including xenobiotics) that gain entry into the organism through dietary food stuffs, for which it has been previously posited that they could represent instances of gene-culture coevolution in humans [13, 15, 20].

Out of those genes*, PLRP2*, *NAT2* and *CYP3A5* were found to present signs in their distribution patterns evoking the action of environmental selective pressures, though of diverse nature and strength.

The most unequivocal signature of selection was associated with *CYP3A5* that displayed a level of inter-population differentiation dramatically surmounting even the most conservative neutral expectations. Contrarily to our starting hypothesis, however, the amount of salt presumed to be ingested across main dietary habits did not accounted for the distribution of *CYP3A5*, which instead was highly determined by the geographical location of populations in the North or in the South of the Tropic of Cancer. So, the analyses here undertaken fully support previous findings indicating that CYP3A5*3 evolved under a selective pressure determined by an environmental factor correlated with latitude [24], but also add accuracy to the interpretation pointing toward a factor shared by regions located above or below the Northern Tropic. *CYP3A5* has been intensively explored in the context of the genetic factors contributing to hypertension susceptibility, known to vary widely across different human populations. Nearly 40 years ago Gleibermann [36] proposed the “sodium retention” hypothesis, according to which the high rate of hypertension in certain populations could partially be due to a genetic background that was environmental adaptive, presuming that efficient salt retaining mechanisms might had been advantageous in the hot savanna climate where humans first emerged. More recently, it was argued that hypertension susceptibility was ancestral in humans, and that differential susceptibility arose due to distinct selective pressures after the Out-of-Africa expansion of modern humans [27]. *CYP3A5* is being often quoted to address the evolutionary perspective of hypertension susceptibility, due to the demonstrated role of *CYP3A5* enzymes in sodium homeostasis, even though the many studies that analyzed the relationship between *CYP3A5* genotypes and blood pressure/hypertension have provided quite inconsistent results (reviewed in Lamba et al. [37]). So, together with the clarification of the link between *CYP3A5* and blood pressure, future lines of research should pay more attention to the role of *CYP3A5* enzymes in the physiological processes related with thermoregulation and/or with neutralization of effects of sunlight exposure. In the highly heat stressful intertropical region, there is a regular need to deal with the threat of dehydration, which may raise complicated physiological responses in wet or dry climates under which the efficient control of heat loss likely differs. Interestingly, the involvement of *CYP3A5* in such responses seems to obtain support from the recent discovery of an osmosensitive transcriptional control of human *CYP3A4*, *CYP3A7*, and *CYP3A5* that revealed increased mRNA expressions under ambient hypertonicity [38].

Concerning *PLRP2*, the explorations here undertaken led in essence to corroborate the findings of Hancock et al. [15], indicating that diversity at the locus is somehow connected with mode of subsistence in populations. In fact, the assessed truncated allele showed to be significantly more frequent in farmers comparatively to groups not relying in farming, with the general trend, inferred from the whole set of African and Eurasian populations, pointing to a clinal decrease in frequency from farmers, next pastoralists towards agriculturalists. In addition, the global differentiation at this variant fell outside the neutral expectations, except when the HIM model was used in the tests for selection in Africa plus Eurasia. Hancock et al. [15] have associated the worldwide distribution of *PLRP2* to the content in cereals in diets of populations, on the grounds of the important role of the protein encoded by *PLRP2* in plant-based diets once, unlike other pancreatic lipases, this enzyme hydrolyzes galactolipids, which are the main triglyceride component in plants [15]. However, the recent demonstration that the truncated allele addressed in their (and our) study exhibits near absence of secretion makes it unlikely that the encoded product may contribute to plant lipid digestion in humans [39], which seemingly undermines the biological basis originally proposed. In the meanwhile, new insights arose on the physiological role of *PLRP2*, suggesting for instance a likely major influence in fat digestion in newborns [40]. This refreshed information opens new perspectives that deserve future investigation to clarify whether cereal content or other *PLRP2* substrate, or amount of substrate, differing in hunter-gatherers, herders and farmers is the factor that exerted the selective pressure contributing to shape the current pattern of *PLRP2* diversity in human populations.

Before, however, it is necessary to overcome the uncertainty raised by the presence of significant LD between *PLRP2* and *PLRP1.* The two genes encode lipases that show high sequence homology and that assumedly participate in dietary fat metabolism, although not being yet clarified their differences in substrate specificity. Consequently, for the moment is not possible to discriminate between which variations at *PLRP2* or *PLRP1* are the best candidates to be causative of the selective signals detected.

With respect to *NAT2*, in agreement with earlier studies [21, 22] we also detected that the average frequency of a slow acetylator allele was statistically lower in hunter-gatherers than in food-producer populations, when Eurasian and African populations were taken as a set. Furthermore, the same slow acetylator variant, which is the widespread NAT2*6 allele, revealed an unusual low level of geographical structure across Africa and Eurasia, indicating that it was subjected to drifting constrains that likely could arise under the action of a mode of selection resulting in such a homogeneous allele distribution. In the tests for selection involving *NAT2*, significant departure from the neutral expectation was captured when the simple IM demographic model was assumed, which can raise uncertainty on whether signs of more subtle selective pressures might mistakenly escape the stringency of the HIM. The adaptive evolution of *NAT2* has been supported by a number of different studies [19-23, 41, 42], including the examination of *NAT2* sequence data whose patterns of diversity made it plausible that slow-acetylating variants have been subject to weak selective pressures [23]. However, it is yet to be clarified the nature of such pressures. Luca et al. [22] tentatively claimed that it could be related with the diminished availability of folates in diet brought with the shift from economies relying in hunting and gathering to those based on farming and herding of domesticates. In line with the hypothesis, very recently a significant correlation was reported between *NAT2* acetylator phenotypes and historical dietary habitudes in India with the slow acetylator prevalence being higher in regions where is higher the proportion of vegetarians populations [43]. A major problem with this folate-related model is the still non-demonstrated role of *NAT2* human enzymes in folate metabolism [44]. Endogenous substrates for human *NAT2* are not known, although being well established that *NAT2* catalyzes the acetylation of many xenobiotics [44]. Since the exposure to xenobiotic substances or to concentration of xenobiotics likewise must had altered along the change in diets experienced by producers of food resources, the role of this kind of substances in shaping diversity at *NAT2* deserves further attention.

In summary, we provided added evidence that diversity at *PLRP2* and *NAT2* harbor signatures of genetic adaptations that might have been triggered by the diversification of modes of subsistence and diets that human populations began to experience after the rise of the Neolithic. Before that, modern humans had started to leave their original homeland in Africa, traveling out of the continent to colonize all regions in the globe. They progressively reached a wide range of new environments, facing new selective pressures that may have contributed to shape human genetic diversity. In *CYP3A5*, the compelling correlation with regions North and South the Tropic of Cancer, makes it likely that it belongs to the yet not fully understood catalog of genetic adaptations triggered by environmental stresses. Furthermore, the genetic signatures that *CYP3A5* harbors seem strength enough to have been driven by very long-lasting selection.

Finally, we were unable to confirm the hypotheses at stake that *AGXT* and *MTRR* could also be diet-selected genes, since their diversity patterns could be well reconciled with demographic history at least of African and Eurasian populations.

## Conclusions

In this study, we found signs that *PLRP2*, *NAT2* and *CYP3A5*, three genes assumedly involved in the metabolism of substances (including xenobiotics) that gain entry into the organism through dietary food stuffs, can represent instances of gene-culture coevolution in humans. Concerning *PLRP2*, it is still needed to clarify whether the signal detected is not a hitchhiking effect of its neighbor *PLRP1*. In addition, it is also necessary to demonstrate which were the biological mechanisms, and the environmental factors involved as well as their interactions, to understand the nature of the selective pressures that contributed to shape current patterns of genetic diversity at those *loci*. Furthermore, functional studies are needed to demonstrate the putative biological impact of the variations assessed, which ultimately would also exclude that the detected signs of selection could be due to other variations in *linkage disequilibrium* with those that were here examined.

## Methods

### Ethics statement

The current study was approved by the Institute of Molecular Pathology and Immunology of the University of Porto institutional review board. All samples involved were anonymised DNA extracts previously obtained from healthy unrelated individuals. The samples were collected under written informed consent. The current study complies with the ethical principles of the 2000 Helsinki Declaration of the 206 World Medical Association (http://jama.jamanetwork.com).

### Samples and DNA extraction

A total amount of 361 individuals from six sub-Saharan African populations with different modes of subsistence were analyzed, i) 144 were farmers/agriculturalists from three populations: 32 from Angola (ANG), 82 from Equatorial Guinea (EQG) and 30 from Mozambique (MOZ); ii) 116 were herders/pastoralists from the Karamoja region in Northern Uganda (UGN); and iii) 101 belonged to two hunter-gatherer/forager populations: 39 Baka Pygmies from Gabon (BPY) and 62 Ju/hoansi from Tsumkwe, a small settlement in North Eastern Namibia (KNA). Forty-eight individuals from the Portuguese population (PTG) were additionally studied. Each population was assigned to a specific mode of subsistence and main diet component according to Murdock Ethnographic Atlas (http://lucy.ukc.ac.uk/cgi-bin/uncgi/Ethnoatlas/atlas.vopts), Encyclopedia of World Cultures, Africa: An Encyclopedia for Students [45].

From blood samples stored in FTA™ cards (Whatman), total DNA was extracted using a standard phenol-chlorophorm protocol [46] in the sample from Equatorial Guinea, while in the remaining samples it was done as described in previous works: Angola [47], Mozambique [48], Uganda [49], Baka Pygmies [50], Khoisan (Marks et al., submitted) and Portugal.

### Genotyping

The screened SNPs were c.32C > T (rs34116584; NM_000030.2 *AGXT* gene), c.1074G > A (rs4751995; NM_005396.3 *PLRP2* gene), c.1130A > G (rs162036; NM_024010.2 *MTRR* gene), c.219-237G > A (rs776746; NM_000777.3 *CYP3A5* gene) and the following 4 variants in *NAT2* gene (NM_000015.2): c.191G > A (rs1801279), c.341 T > C (rs1801280), c.590G > A (rs1799930) and c.857G > A (rs1799931). The last 4 SNPS, defining the alleles NAT2*14, NAT2*5, NAT2*6 and NAT2*7, respectively, were selected in this study because reportedly they allow inferring the acetylator phenotypes with high accuracy [41].

A pair of primers was designed for each SNP using Primer3 software ver. 4.0. Possible secondary structures or interactions between primers were checked with AutoDimer software ver. 1.0 [51] (Additional file 11: Table S4). A multiplex Polymerase Chain Reaction (PCR) system was developed to co-amplify the regions containing the eight SNPs. The genotyping methodology was based in a multiplex minisequencing reaction, using the Single Base Extension (SBE) [31] reaction kit (Applied Biosystems). SBE primers were designed and tested identically as described above. Poly tails of varying lengths were attached to the 5′ end of each primer in order to avoid identical fragment sizes, allowing simultaneous typing of multiple variants in the same reaction (Additional file 12: Table S5). SBE products were run on ABI 3130 Genetic Analyser (AB Applied Biosystems) and the electropherograms were analyzed using GeneMapper software ver. 4.0. (Applied Biosystems, Foster City, USA), based on fragment size inferred with GeneScan-120 size standard.

Chromosomal locations and genomic segments from the 5 genes were obtained using the latest version of the human genome assembly GRCh37 (http://www.ensembl.org/).

### Statistical analysis

The Arlequin software ver. 3.5.1 [52] was used to estimate allele and haplotype frequencies, to test for Hardy-Weinberg Equilibrium (HWE) and to calculate genetic distances (*F*_ST_). Regarding the *NAT2* gene, to account for *linkage disequilibrium* (LD) between SNPs, from the unphased multilocus genotypic data, haplotypic frequencies defined by the 4 SNPs were computationally estimated also with Arlequin software ver. 3.5.1. [52] and the acetylation phenotypes deduced from the pair of haplotypes carried by each subject [41].

In order to determine whether means of allele frequencies were statistically different between groups of populations defined according to various criteria, One-Way ANOVA tests were performed. The Spearman Rank Correlation Score Corrected (SRCSC) was used to evaluate the correlation between allele frequencies and latitude. Both statistical analyses were performed on the website for statistical computation VassarStats (http://vassarstats.net).

The graphical representation of a *F*_ST_ distance matrix was constructed by means of the Multidimensional Scaling (MDS) procedure implemented in StatSoft, Inc. (2007), Statistica version 8.0 (www.statsoft.com).

To investigate possible signals of selection we obtained the neutral distribution of *F*_ST_s conditional on heterozygozity based on genotypic data from a validated panel of 52 assumedly neutral SNPs for human identification [53], using the 61 African and Eurasian populations contained in the SNP*for*ID browser, to draw a neutral dispersion cloud 50,000 coalescent simulations of 100 demes were carried on Arlequin software. For variants at *AGXT*, *PLRP2*, *MTRR*, *NAT2* and *CYP3A5, F*_ST,_ average heterozygosity within populations (*Het*) and the scaled heterozygosity *Het*/(1-*F*_ST_) were also computed. The null sampling distribution of the empirical *F*_ST_ values was calculated using two distinct models, both implemented in Arlequin software ver. 3.5.1 [52]: i) the classical Island Model at migration-drift equilibrium (IM), conventionally referred to as Fdist approach, proposed by [33], considering together all populations; and ii) the Hierarchical Island Model (HIM) more recently recommended by Excoffier and Hofer [35], for which populations were clustered in 5 groups (Europe, Africa, Middle East, South and East Asia) when a worldwide scale was assumed, or in 4 groups (Northern, Southern, Western and Eastern Africa) when only Africa was considered. Since *F*_ST_ strongly correlates with heterozygosity [33], the empirical *P*-value for each locus was calculated within bins of 2,0000 SNPs grouped according to Minimum Allele Frequency (MAF), as the proportion of the bivariate probability distribution which was less probable than the estimated values, in the same way as calculated in the DFdist software package [33].

Hierarchical Analysis of Molecular Variance (AMOVA) was performed in the Arlequin software ver. 3.5.1. defining population groups according to: i) mode of subsistence, ii) main diet component, and iii) geography in the context of Eurasia and Africa. Additionally for *CYP3A5,* populations groups were also defined according to latitude and location North or South the Tropic of Cancer.

Finally for LD patterns analyses, the .ped files containing the data sets were first manipulated with gPLINK software (http://pngu.mgh.harvard.edu/purcell/plink/) [54] and then exported to Haploview software ver. 4.1 [55] to calculate *D’* (normalized *D,* where *D’* = *D*/*D*max measures the LD strength) and *r*^*2*^ (squared correlation coefficient measure of LD between the two *loci*) parameters and visualize the LD plots, considering a window of 200, 300 and 500 Kb for *PLRP2*, *CYP3A5* and *NAT2* genes, respectively.

### Comparative data

Viewing comparative analyses, data for other populations were retrieved from the database dbCLINE (http://genapps.uchicago.edu/software.html) from the Di Rienzo laboratory, which includes information from the International HaPMap Project Phase III (http://hapmap.ncbi.nlm.nih.gov/), the Human Genome Diversity Project Panel-Centre d’Etude du Polymorphisme Humain (HGDP-CEPH); and also we included data already published in other works [56-59]. The populations’ abbreviations used in Figs. 1 and 2 are MAN (Mandenka), NBT (North Bantu), MOB (Mozabite), SBT (South Bantu), LUH (Luhya), TIG (Tigray), AMH1 (Amhara1), AFA (Afar), YOR (Yoruba), AMH2 (Amhara2), ARC (Ari Cultivator), WOL (Wolayta), ANU (Anuak), ARB (Ari Blacksmith), JPT (Japanese), NAX (Naxi), LAH (Lahu), MIA (Miaozu), TUJ (Tujia), SHE (She), YIZ (Yizu), CAM (Cambodian), HAN (Han), DAU (Daur), DAI (Dai), BER (Bergamo), RUS (Russian), TSC1 (Tuscan1), SAR (Sardinian), FRC (French), BAS (Basque), TUSC2 (Tuscan2), ORC (Orcadian), ADY (Adygei), DRU (Druze), PAL (Palestinian) KAL (Kalash), PAT (Pathan), GUJ (Gujarati), BUR (Burusho), SIN (Sindhi), XIB (Xibo), GUM (Gumuz), SOM (Somali), MAS (Maasai), MON (Mongola), YAK (Yakut), BAL (Balochi), MAK (Makrani), TU (Tu), UYG (Uygur), BED (Bedouin), BRA (Brahui), HAZ (Hazara), SAN (San), BIP (Biaka Pygmies), KUV (Kung Vasekela), MBP (Mbuti Pygmies) KHO1 (Khomani2), SAD (Sadawe), HAD (Hadza), HEZ (Hezhen), NYU (Naukan Yup’ik), ORO (Orogen) and MCH (Maritime Chukchee). More detailed information is summarized in Additional file 2: Table S2.

## Additional files

Additional file 1: Table S1. Hardy-Weinberg Equilibrium test. Additional file 2: Table S2. Detailed data on mode of subsistence, diet and geography of the populations used for comparative analyses. Additional file 3: Table S3. Haplotype and phenotype frequencies for *NAT2* gene. Additional file 4: Figure S1. LD patterns for *PLRP2* in African populations from Pagani et al. (2012) [58]. The studied SNP is indicated by a red rectangle. In black triangles are represented the LD blocks. The degree of LD between pairs of markers is indicated by the |D’| statistic (|D’| = 1, red; |D’| < 1, shades of red). Additional file 5: Figure S2. LD patterns for *PLRP2* in African populations from Henn et al. (2011) [57]. The studied SNP is indicated by a red rectangle. In black triangles are represented the LD blocks. The degree of LD between pairs of markers is indicated by the |D’| statistic (|D’| = 1, red; |D’| < 1, shades of red). Additional file 6: Figure S3. LD patterns for *PLRP2* in African populations from Schlebusch et al. (2012) [59]. The studied SNP is indicated by a red rectangle. In black triangles are represented the LD blocks. The degree of LD between pairs of markers is indicated by the |D’| statistic (|D’| = 1, red; |D’| < 1, shades of red). Additional file 7: Figure S4. LD patterns for *NAT2* in African populations from Henn et al. (2011) [57]. A red arrow indicates the studied SNP. In black triangles are represented the LD blocks. The degree of LD between pairs of markers is indicated by the |D’| statistic (|D’| = 1, red; |D’| < 1, shades of red). Additional file 8: Figure S5. LD patterns for *CYP3A5* in African populations from Pagani et al. (2012) [58]. The studied SNP is indicated by a red rectangle. In black triangles are represented the LD blocks. The degree of LD between pairs of markers is indicated by the |D’| statistic (|D’| = 1, red; |D’| < 1, shades of red). Additional file 9: Figure S6. LD patterns for *CYP3A5* in African populations from Henn et al. (2011) [57]. The studied SNP is indicated by a red rectangle. In black triangles are represented the LD blocks. The degree of LD between pairs of markers is indicated by the |D’| statistic (|D’| = 1, red; |D’| < 1, shades of red). Additional file 10: Figure S7. LD patterns for *CYP3A5* in African populations from Schlebusch et al. (2012) [59]. The studied SNP is indicated by a red rectangle. In black triangles are represented the LD blocks. The degree of LD between pairs of markers is indicated by the |D’| statistic (|D’| = 1, red; |D’| < 1, shades of red). Additional file 11: Table S4. Amplification primers sequences. Additional file 12: Table S5. Mini-sequencing primers sequences.
