# Supplementary material for: Exploring the relationship between lifestyles, diets and genetic adaptations in humans
Source: BMC Genet. 2015 May 28;16:55. doi: 10.1186/s12863-015-0212-1 (PMC4445807; doi:10.1186/s12863-015-0212-1)
Supplement: Additional file 1: Table S1. — Hardy-Weinberg Equilibrium test. [file 12863_2015_212_MOESM1_ESM.pdf]

Table S1. Hardy-Weinberg Equilibrium test.

	**c.32C>T (*AGXT*)**			**c.1074G>A (*PLRP2*)**			**c.1130A>G (*MTRR*)**			**c.191G>A (*NAT2*14*)**			**c.341T>C (*NAT2*5*)**			**c.590G>A (*NAT2*6*)**			**c.857G>A (*NAT2*7*)**			**c.219-237G>A (*CYP3A5*)**		
**POPULATION**	H_{OB}	H_{EX}	***P*-value**	H_{OB}	H_{EX}	***P*-value**	H_{OB}	H_{EX}	***P*-value**	H_{OB}	H_{EX}	***P*-value**	H_{OB}	H_{EX}	***P*-value**	H_{OB}	H_{EX}	***P*-value**	H_{OB}	H_{EX}	***P*-value**	H_{OB}	H_{EX}	***P*-value**
**ANG (n=32)**		*		0.5652	0.4493	0.3462	0.7059	0.5134	0.1616	0.0435	0.2638	0.0014	0.1364	0.3330	0.0171	0.1818	0.47357	0.0055		*		0.4000	0.3722	1.0000
**EQG (n=82)**	0.0964	0.0923	1.0000	0.4048	0.4388	0.6165	0.5287	0.4614	0.2430	0.1954	0.1773	1.0000	0.5529	0.4629	0.0972	0.3333	0.29512	0.4487	0.0465	0.0457	1.0000	0.2143	0.2464	0.3581
**MOZ (n=30)**	0.0741	0.0727	1.0000	0.3333	0.3638	0.6319	0.4333	0.5034	0.4818	0.2857	0.2509	1.0000	0.4000	0.3846	1.0000	0.4762	0.41812	0.6322		*		0.2333	0.2096	1.0000
**UGN (n=116)**	0.0909	0.1355	0.0099	0.4404	0.4799	0.4249	0.5534	0.4752	0.0994	0.1183	0.1307	0.3621	0.3902	0.4788	0.1077	0.4255	0.42895	1.0000	0.0110	0.0110	1.0000	0.4112	0.3598	0.1783
**BPY (n=39)**	0.0294	0.0294	1.0000	0.3784	0.3110	0.3089	0.5128	0.4795	0.7428	0.0526	0.0519	1.0000	0.2105	0.3046	0.0838	0.4595	0.38689	0.3926		*		0.2895	0.2509	1.0000
**KNA (n=62)**		*		0.0161	0.0476	0.0241	0.2742	0.2385	0.5856		*		0.1312	0.1236	1.0000	0.0968	0.09284	1.0000	0.1613	0.1762	0.4448	0.3871	0.3341	0.2703
**PTG (n=48)**	0.2979	0.3130	0.6605	0.4681	0.5052	0.7704	0.2340	0.2409	1.0000		*		0.3913	0.5055	0.1465	0.4043	0.32601	0.1708	0.0638	0.1018	0.1051	0.1522	0.1785	0.3510

H_{OB} – Heterozygozity observed; H_{EX} – Heterozygozity expected; * – this locus is monomorphic. Significant differences, after Bonferroni's correction for multiple tests are highlighted in **bold**; populations' abbreviations as referred in material and methods section.
