# Supplementary material for: Exploring the relationship between lifestyles, diets and genetic adaptations in humans
Source: BMC Genet. 2015 May 28;16:55. doi: 10.1186/s12863-015-0212-1 (PMC4445807; doi:10.1186/s12863-015-0212-1)
Supplement: Additional file 2: Table S2. — Detailed data on mode of subsistence, diet and geography of the populations used for comparative analyses. [file 12863_2015_212_MOESM2_ESM.pdf]

Table S2. Detailed data on mode of subsistence, diet and geography of the populations used for comparative analyses.

		AMOVA groups classification						
	Population	n	*mode of subsistence*	*main diet component*	*geography*	*latitude*	*intertropical zone*	reference
c.32C>T (AGXT)	Angola	29	agriculturalist	meat-poor diet	Africa	*NA*	*NA*	*this study*
	Mozambique	30	agriculturalist	meat-poor diet	Africa	*NA*	*NA*	*this study*
	Equatorial Guinea	87	agriculturalist	meat-poor diet	Africa	*NA*	*NA*	*this study*
	Nigeria	62	agriculturalist	meat-poor diet	Africa	*NA*	*NA*	Caldwell *et al* (2004)
	Ethiopia	69	agriculturalist	meat-poor diet	Africa	*NA*	*NA*	Thomas *et al* (2002)
	Portugal	47	agriculturalist	meat-poor diet	Europe	*NA*	*NA*	*this study*
	North Wales	82	agriculturalist	meat-poor diet	Europe	*NA*	*NA*	Caldwell *et al* (2004)
	Turkey (Anatolia)	88	agriculturalist	meat-poor diet	Europe	*NA*	*NA*	Thomas *et al* (2003)
	Armenia	73	agriculturalist	meat-poor diet	Europe	*NA*	*NA*	Caldwell *et al* (2004)
	Norway	76	agriculturalist	meat-poor diet	Europe	*NA*	*NA*	Weale *et al* (2002)
	Ashkenazi Jews (Germany)	73	agriculturalist	meat-poor diet	Europe	*NA*	*NA*	Thomas *et al* (2002)
	India (Mombai)	84	agriculturalist	meat-poor diet	South Asia	*NA*	*NA*	Thomas *et al* (2002)
	Tajiks (Fergana)	17	agriculturalist	meat-poor diet	East Asia	*NA*	*NA*	Ségurel *et al* (2010)
	Tajiks (Gharm)	23	agriculturalist	meat-poor diet	East Asia	*NA*	*NA*	Ségurel *et al* (2010)
	Tajiks (Pejinkent)	24	agriculturalist	meat-poor diet	East Asia	*NA*	*NA*	Ségurel *et al* (2010)
	Tajiks (Douchambe)	26	agriculturalist	meat-poor diet	East Asia	*NA*	*NA*	Ségurel *et al* (2010)
	Sichuan China	86	agriculturalist	meat-poor diet	East Asia	*NA*	*NA*	Caldwell *et al* (2004)
	Uganda	110	pastoralist	meat-poor diet	Africa	*NA*	*NA*	*this study*
	Kasaks (Karakalpakia)	30	pastoralist	meat-rich diet	East Asia	*NA*	*NA*	Ségurel *et al* (2010)
	Mongolia	80	pastoralist	meat-rich diet	East Asia	*NA*	*NA*	Caldwell *et al* (2004)
	Kyrgyz1 (Narin)	20	pastoralist	meat-rich diet	East Asia	*NA*	*NA*	Ségurel *et al* (2010)
	Kasaks (Buraka)	49	pastoralist	meat-rich diet	East Asia	*NA*	*NA*	Ségurel *et al* (2010)
	Kyrgyz2 (Narin)	26	pastoralist	meat-rich diet	East Asia	*NA*	*NA*	Ségurel *et al* (2010)
	Turkmen (Karakalpakia)	34	pastoralist	meat-rich diet	East Asia	*NA*	*NA*	Ségurel *et al* (2010)
	Kyrgyz (Andijan)	32	pastoralist	meat-rich diet	East Asia	*NA*	*NA*	Ségurel *et al* (2010)
	Karakalpaks	30	pastoralist	meat-rich diet	East Asia	*NA*	*NA*	Ségurel *et al* (2010)
	Saami (Sweden)	34	pastoralist	meat-rich diet	Europe	*NA*	*NA*	Thomas *et al* (2002)
	Khoisan Namibia	62	hunter-gatherer	meat-rich diet	Africa	*NA*	*NA*	*this study*
	Baka Pygmies	39	hunter-gatherer	meat-rich diet	Africa	*NA*	*NA*	*this study*
	Khomani Bushman San (B)	35	hunter-gatherers	meat-rich	Africa	*NA*	*NA*	Henn *et al* (2011)
	Hadza (Tanzania)	20	hunter-gatherers	meat-rich	Africa	*NA*	*NA*	Henn *et al* (2011)
	Sandawe (Tanzania)	35	hunter-gatherers	meat-rich	Africa	*NA*	*NA*	Henn *et al* (2011)
	Angola	29	agriculturalist	specialized in cereals	Africa	*NA*	*NA*	*this study*
	Mozambique	30	agriculturalist	specialized in cereals	Africa	*NA*	*NA*	*this study*
	Equatorial Guinea	87	agriculturalist	specialized in cereals	Africa	*NA*	*NA*	*this study*
	N. Bantu	12	agriculturalist	specialized in cereals	Africa	*NA*	*NA*	HGDP-CEPH
	Mandenka	24	agriculturalist	specialized in cereals	Africa	*NA*	*NA*	HGDP-CEPH
	S. Bantu	8	agriculturalist	specialized in cereals	Africa	*NA*	*NA*	HGDP-CEPH
	Luhya	88	agriculturalist	specialized in cereals	Africa	*NA*	*NA*	HapMap Ph3
	Mozabite	30	agriculturalist	specialized in cereals	Africa	*NA*	*NA*	HGDP-CEPH
	Yoruba	25	agriculturalist	specialized in cereals	Africa	*NA*	*NA*	HGDP-CEPH
	Portugal	47	agriculturalist	specialized in cereals	Europe	*NA*	*NA*	*this study*
	Basque	24	agriculturalist	specialized in cereals	Europe	*NA*	*NA*	HGDP-CEPH
	Bergamo	14	agriculturalist	specialized in cereals	Europe	*NA*	*NA*	HGDP-CEPH
	French	29	agriculturalist	specialized in cereals	Europe	*NA*	*NA*	HGDP-CEPH
	Orcadian	16	agriculturalist	specialized in cereals	Europe	*NA*	*NA*	HGDP-CEPH
	Russian	25	agriculturalist	specialized in cereals	Europe	*NA*	*NA*	HGDP-CEPH
	Sardinian	28	agriculturalist	specialized in cereals	Europe	*NA*	*NA*	HGDP-CEPH
	Tuscan1	8	agriculturalist	specialized in cereals	Europe	*NA*	*NA*	HGDP-CEPH
	Tuscan2	89	agriculturalist	specialized in cereals	Europe	*NA*	*NA*	HapMap Ph3
	Adygei	17	agriculturalist	specialized in cereals	Middle Eastern	*NA*	*NA*	HGDP-CEPH
	Druze	48	agriculturalist	specialized in cereals	Middle Eastern	*NA*	*NA*	HGDP-CEPH
	Palestinian	51	agriculturalist	specialized in cereals	Middle Eastern	*NA*	*NA*	HGDP-CEPH
	Burusho	25	agriculturalist	specialized in cereals	South Asia	*NA*	*NA*	HGDP-CEPH
	Kalash	25	agriculturalist	specialized in cereals	South Asia	*NA*	*NA*	HGDP-CEPH
	Pathan	25	agriculturalist	specialized in cereals	South Asia	*NA*	*NA*	HGDP-CEPH
	Sindhi	25	agriculturalist	specialized in cereals	South Asia	*NA*	*NA*	HGDP-CEPH
	Xibo	9	agriculturalist	specialized in cereals	South Asia	*NA*	*NA*	HGDP-CEPH
	Gujarati	88	agriculturalist	specialized in cereals	South Asia	*NA*	*NA*	HapMap Ph3
	Cambodian	11	agriculturalist	specialized in cereals	East Asia	*NA*	*NA*	HGDP-CEPH
	Dai	10	agriculturalist	specialized in cereals	East Asia	*NA*	*NA*	HGDP-CEPH
	Han	45	agriculturalist	specialized in cereals	East Asia	*NA*	*NA*	HGDP-CEPH
	Japanese	31	agriculturalist	specialized in cereals	East Asia	*NA*	*NA*	HGDP-CEPH

c.1074G>A (PLRP2)	Lahu	10	agriculturalist	specialized in cereals	East Asia	*NA*	*NA*	HGDP-CEPH
	Miaozu	10	agriculturalist	specialized in cereals	East Asia	*NA*	*NA*	HGDP-CEPH
	Naxi	10	agriculturalist	specialized in cereals	East Asia	*NA*	*NA*	HGDP-CEPH
	She	10	agriculturalist	specialized in cereals	East Asia	*NA*	*NA*	HGDP-CEPH
	Tujia	10	agriculturalist	specialized in cereals	East Asia	*NA*	*NA*	HGDP-CEPH
	Yizu	10	agriculturalist	specialized in cereals	East Asia	*NA*	*NA*	HGDP-CEPH
	Daur	10	agriculturalist	specialized in cereals	East Asia	*NA*	*NA*	HGDP-CEPH
	Afar (Ethiopia)	12	agriculturalist	specialized in cereals	Africa	*NA*	*NA*	Pagani *et al* (2012)
	Amhara1	*NA*	agriculturalist	not specialized in cereals	Africa	*NA*	*NA*	HapMap Ph3
	Amhara2	26	agriculturalist	specialized in cereals	Africa	*NA*	*NA*	Pagani *et al* (2012)
	Anuak (Ethiopia)	23	agriculturalist	specialized in cereals	Africa	*NA*	*NA*	Pagani *et al* (2012)
	Ari Blacksmith (Ethiopia)	17	agriculturalist	specialized in cereals	Africa	*NA*	*NA*	Pagani *et al* (2012)
	Ari cultivator (Ethiopia)	24	agriculturalist	specialized in cereals	Africa	*NA*	*NA*	Pagani *et al* (2012)
	Tigray (Ethiopia)	21	agriculturalist	specialized in cereals	Africa	*NA*	*NA*	Pagani *et al* (2012)
	Wolayta (Ethiopia)	8	agriculturalist	specialized in cereals	Africa	*NA*	*NA*	Pagani *et al* (2012)
	SEBantu (Herero_Namibia)	12	agriculturalist	specialized in cereals	Africa	*NA*	*NA*	Schlebusch *et al* (2012)
	SWBantu (South Africa)	20	agriculturalist	specialized in cereals	Africa	*NA*	*NA*	Schlebusch *et al* (2012)
	Uganda	110	pastoralist	not specialized in cereals	Africa	*NA*	*NA*	*this study*
	Masaai	143	pastoralist	not specialized in cereals	Africa	*NA*	*NA*	HapMap Ph3
	Bedouin	49	pastoralist	not specialized in cereals	South Asia	*NA*	*NA*	HGDP-CEPH
	Brahui	25	pastoralist	not specialized in cereals	South Asia	*NA*	*NA*	HGDP-CEPH
	Hazara	25	pastoralist	not specialized in cereals	South Asia	*NA*	*NA*	HGDP-CEPH
	Makrani	25	pastoralist	not specialized in cereals	South Asia	*NA*	*NA*	HGDP-CEPH
	Uygur	10	pastoralist	not specialized in cereals	South Asia	*NA*	*NA*	HGDP-CEPH
	Tu	10	pastoralist	not specialized in cereals	South Asia	*NA*	*NA*	HGDP-CEPH
	Mongola	10	pastoralist	not specialized in cereals	East Asia	*NA*	*NA*	HGDP-CEPH
	Balochi	25	pastoralist	not specialized in cereals	East Asia	*NA*	*NA*	HGDP-CEPH
	Yakut	25	pastoralist	not specialized in cereals	East Asia	*NA*	*NA*	HGDP-CEPH
	Somali (Ethiopia)	17	pastoralist	not specialized in cereals	Africa	*NA*	*NA*	Pagani *et al* (2012)
	Gumuz (Ethiopia)	19	pastoralist	not specialized in cereals	Africa	*NA*	*NA*	Pagani *et al* (2012)
	Nama (Namibia)	20	pastoralist	not specialized in cereals	Africa	*NA*	*NA*	Schlebusch *et al* (2012)
	Uganda	110	pastoralist	not specialized in cereals	Africa	*NA*	*NA*	*this study*
	Masaai	143	pastoralist	not specialized in cereals	Africa	*NA*	*NA*	HapMap Ph3
	Oroqen	10	hunter-gatherer	not specialized in cereals	East Asia	*NA*	*NA*	HGDP-CEPH
	Hezhen	10	hunter-gatherer	not specialized in cereals	East Asia	*NA*	*NA*	HGDP-CEPH
	Maritime Chukchee	*NA*	hunter-gatherer	not specialized in cereals	East Asia	*NA*	*NA*	HGDP-CEPH
	Naukan Yup'ik	*NA*	hunter-gatherer	not specialized in cereals	East Asia	*NA*	*NA*	HGDP-CEPH
	Khoisan Namibia	62	hunter-gatherer	not specialized in cereals	Africa	*NA*	*NA*	*this study*
	San	7	hunter-gatherer	not specialized in cereals	Africa	*NA*	*NA*	HGDP-CEPH
	Baka Pygmies	39	hunter-gatherer	not specialized in cereals	Africa	*NA*	*NA*	*this study*
	Biaka Pygmy	36	hunter-gatherer	not specialized in cereals	Africa	*NA*	*NA*	HGDP-CEPH
	Mbuti Pygmy	15	hunter-gatherer	not specialized in cereals	Africa	*NA*	*NA*	HGDP-CEPH
	Khomani2 (South Africa)	35	hunter-gatherers	not specialized in cereals	Africa	*NA*	*NA*	Henn *et al* (2011)
	Hadza (Tanzania)	20	hunter-gatherers	not specialized in cereals	Africa	*NA*	*NA*	Henn *et al* (2011)
	Sandawe (Tanzania)	35	hunter-gatherers	not specialized in cereals	Africa	*NA*	*NA*	Henn *et al* (2011)
	Khwe (Angola)	17	hunter-gatherers	not specialized in cereals	Africa	*NA*	*NA*	Schlebusch *et al* (2012)
	!Xun (Angola)	19	hunter-gatherers	not specialized in cereals	Africa	*NA*	*NA*	Schlebusch *et al* (2012)
	Gui and Ghana (Botswana)	15	hunter-gatherers	not specialized in cereals	Africa	*NA*	*NA*	Schlebusch *et al* (2012)
	Ju/'hoansi (Namibia)	18	hunter-gatherers	not specialized in cereals	Africa	*NA*	*NA*	Schlebusch *et al* (2012)
	Karretjie (South Africa)	20	hunter-gatherers	not specialized in cereals	Africa	*NA*	*NA*	Schlebusch *et al* (2012)
	Khomani2 (South Africa)	39	hunter-gatherers	not specialized in cereals	Africa	*NA*	*NA*	Schlebusch *et al* (2012)
	Kung Vasekela	*NA*	hunter-gatherers	not specialized in cereals	Africa	*NA*	*NA*	HGDP-CEPH
	Angola	29	agriculturalist	folate poor-diet	Africa	*NA*	*NA*	*this study*
	Mozambique	30	agriculturalist	folate poor-diet	Africa	*NA*	*NA*	*this study*
	Equatorial Guinea	87	agriculturalist	folate poor-diet	Africa	*NA*	*NA*	*this study*
	N. Bantu	12	agriculturalist	folate poor-diet	Africa	*NA*	*NA*	HGDP-CEPH
	Mandenka	24	agriculturalist	folate poor-diet	Africa	*NA*	*NA*	HGDP-CEPH
	S. Bantu	8	agriculturalist	folate poor-diet	Africa	*NA*	*NA*	HGDP-CEPH
	Luhya	88	agriculturalist	folate poor-diet	Africa	*NA*	*NA*	HapMap Ph3
	Mozabite	30	agriculturalist	folate poor-diet	Africa	*NA*	*NA*	HGDP-CEPH
	Yoruba	25	agriculturalist	folate poor-diet	Africa	*NA*	*NA*	HGDP-CEPH
	Portugal	47	agriculturalist	folate poor-diet	Europe	*NA*	*NA*	*this study*
	Basque	24	agriculturalist	folate poor-diet	Europe	*NA*	*NA*	HGDP-CEPH
	Bergamo	14	agriculturalist	folate poor-diet	Europe	*NA*	*NA*	HGDP-CEPH
	French	29	agriculturalist	folate poor-diet	Europe	*NA*	*NA*	HGDP-CEPH
	Orcadian	16	agriculturalist	folate poor-diet	Europe	*NA*	*NA*	HGDP-CEPH
	Russian	25	agriculturalist	folate poor-diet	Europe	*NA*	*NA*	HGDP-CEPH
	Sardinian	28	agriculturalist	folate poor-diet	Europe	*NA*	*NA*	HGDP-CEPH

c.1130A>G (MTRR)	Tuscan1	8	agriculturalist	folate poor-diet	Europe	*NA*	*NA*	HGDP-CEPH
	Tuscan2	89	agriculturalist	folate poor-diet	Europe	*NA*	*NA*	HapMap Ph3
	Adygei	17	agriculturalist	folate poor-diet	Middle Eastern	*NA*	*NA*	HGDP-CEPH
	Druze	48	agriculturalist	folate poor-diet	Middle Eastern	*NA*	*NA*	HGDP-CEPH
	Palestinian	51	agriculturalist	folate poor-diet	Middle Eastern	*NA*	*NA*	HGDP-CEPH
	Burusho	25	agriculturalist	folate poor-diet	South Asia	*NA*	*NA*	HGDP-CEPH
	Kalash	25	agriculturalist	folate poor-diet	South Asia	*NA*	*NA*	HGDP-CEPH
	Pathan	25	agriculturalist	folate poor-diet	South Asia	*NA*	*NA*	HGDP-CEPH
	Sindhi	25	agriculturalist	folate poor-diet	South Asia	*NA*	*NA*	HGDP-CEPH
	Xibo	9	agriculturalist	folate poor-diet	South Asia	*NA*	*NA*	HGDP-CEPH
	Gujarati	88	agriculturalist	folate poor-diet	South Asia	*NA*	*NA*	HapMap Ph3
	Cambodian	11	agriculturalist	folate poor-diet	East Asia	*NA*	*NA*	HGDP-CEPH
	Dai	10	agriculturalist	folate poor-diet	East Asia	*NA*	*NA*	HGDP-CEPH
	Han	45	agriculturalist	folate poor-diet	East Asia	*NA*	*NA*	HGDP-CEPH
	Japanese	31	agriculturalist	folate poor-diet	East Asia	*NA*	*NA*	HGDP-CEPH
	Lahu	10	agriculturalist	folate poor-diet	East Asia	*NA*	*NA*	HGDP-CEPH
	Miaozu	10	agriculturalist	folate poor-diet	East Asia	*NA*	*NA*	HGDP-CEPH
	Naxi	10	agriculturalist	folate poor-diet	East Asia	*NA*	*NA*	HGDP-CEPH
	She	10	agriculturalist	folate poor-diet	East Asia	*NA*	*NA*	HGDP-CEPH
	Tujia	10	agriculturalist	folate poor-diet	East Asia	*NA*	*NA*	HGDP-CEPH
	Yizu	10	agriculturalist	folate poor-diet	East Asia	*NA*	*NA*	HGDP-CEPH
	Daur	10	agriculturalist	folate poor-diet	East Asia	*NA*	*NA*	HGDP-CEPH
	Afar (Ethiopia)	12	agriculturalist	folate poor-diet	Africa	*NA*	*NA*	Pagani *et al* (2012)
	Amhara1	*NA*	agriculturalist	folate poor-diet	Africa	*NA*	*NA*	HapMap Ph3
	Amhara2 (Ethiopia)	26	agriculturalist	folate poor-diet	Africa	*NA*	*NA*	Pagani *et al* (2012)
	Anuak (Ethiopia)	23	agriculturalist	folate poor-diet	Africa	*NA*	*NA*	Pagani *et al* (2012)
	Ari Blacksmith (Ethiopia)	17	agriculturalist	folate poor-diet	Africa	*NA*	*NA*	Pagani *et al* (2012)
	Ari cultivator (Ethiopia)	24	agriculturalist	folate poor-diet	Africa	*NA*	*NA*	Pagani *et al* (2012)
	Tigray (Ethiopia)	21	agriculturalist	folate poor-diet	Africa	*NA*	*NA*	Pagani *et al* (2012)
	Wolayta (Ethiopia)	8	agriculturalist	folate poor-diet	Africa	*NA*	*NA*	Pagani *et al* (2012)
	SEBantu (Herero_Namibia)	12	agriculturalist	folate poor-diet	Africa	*NA*	*NA*	Schlebusch *et al* (2012)
	SWBantu (South Africa)	20	agriculturalist	folate poor-diet	Africa	*NA*	*NA*	Schlebusch *et al* (2012)
	Uganda	110	pastoralist	folate poor-diet	Africa	*NA*	*NA*	*this study*
	Masaai	143	pastoralist	folate poor-diet	Africa	*NA*	*NA*	HapMap Ph3
	Bedouin	49	pastoralist	folate poor-diet	South Asia	*NA*	*NA*	HGDP-CEPH
	Brahui	25	pastoralist	folate poor-diet	South Asia	*NA*	*NA*	HGDP-CEPH
	Hazara	25	pastoralist	folate poor-diet	South Asia	*NA*	*NA*	HGDP-CEPH
	Makrani	25	pastoralist	folate poor-diet	South Asia	*NA*	*NA*	HGDP-CEPH
	Uygur	10	pastoralist	folate poor-diet	South Asia	*NA*	*NA*	HGDP-CEPH
	Tu	10	pastoralist	folate poor-diet	South Asia	*NA*	*NA*	HGDP-CEPH
	Mongola	10	pastoralist	folate poor-diet	East Asia	*NA*	*NA*	HGDP-CEPH
	Balochi	25	pastoralist	folate poor-diet	East Asia	*NA*	*NA*	HGDP-CEPH
	Yakut	25	pastoralist	folate poor-diet	East Asia	*NA*	*NA*	HGDP-CEPH
	Somali (Ethiopia)	17	pastoralist	folate poor-diet	Africa	*NA*	*NA*	Pagani *et al* (2012)
	Gumuz (Ethiopia)	19	pastoralist	folate poor-diet	Africa	*NA*	*NA*	Pagani *et al* (2012)
	Nama (Namibia)	20	pastoralist	folate poor-diet	Africa	*NA*	*NA*	Schlebusch *et al* (2012)
	Uganda	110	pastoralist	folate poor-diet	Africa	*NA*	*NA*	*this study*
	Masaai	143	pastoralist	folate poor-diet	Africa	*NA*	*NA*	HapMap Ph3
	Oroqen	10	hunter-gatherer	folate rich-diet	East Asia	*NA*	*NA*	HGDP-CEPH
	Hezhen	10	hunter-gatherer	folate rich-diet	East Asia	*NA*	*NA*	HGDP-CEPH
	Maritime Chukchee	*NA*	hunter-gatherer	folate rich-diet	East Asia	*NA*	*NA*	HGDP-CEPH
	Naukan Yup'ik	*NA*	hunter-gatherer	folate rich-diet	East Asia	*NA*	*NA*	HGDP-CEPH
	Khoisan Namibia	62	hunter-gatherer	folate rich-diet	Africa	*NA*	*NA*	*this study*
	San	7	hunter-gatherer	folate rich-diet	Africa	*NA*	*NA*	HGDP-CEPH
	Baka Pygmies	39	hunter-gatherer	folate rich-diet	Africa	*NA*	*NA*	*this study*
	Biaka Pygmy	36	hunter-gatherer	folate rich-diet	Africa	*NA*	*NA*	HGDP-CEPH
	Mbuti Pygmy	15	hunter-gatherer	folate rich-diet	Africa	*NA*	*NA*	HGDP-CEPH
	Khomani1 (South Africa)	35	hunter-gatherers	folate rich-diet	Africa	*NA*	*NA*	Henn *et al* (2011)
	Hadza (Tanzania)	20	hunter-gatherers	folate rich-diet	Africa	*NA*	*NA*	Henn *et al* (2011)
	Sandawe (Tanzania)	35	hunter-gatherers	folate rich-diet	Africa	*NA*	*NA*	Henn *et al* (2011)
	Khwe (Angola)	17	hunter-gatherers	folate rich-diet	Africa	*NA*	*NA*	Schlebusch *et al* (2012)
	!Xun (Angola)	19	hunter-gatherers	folate rich-diet	Africa	*NA*	*NA*	Schlebusch *et al* (2012)
	Gui and Ghana (Botswana)	15	hunter-gatherers	folate rich-diet	Africa	*NA*	*NA*	Schlebusch *et al* (2012)
	Ju/’hoansi (Namibia)	18	hunter-gatherers	folate rich-diet	Africa	*NA*	*NA*	Schlebusch *et al* (2012)
	Karretjie (South Africa)	20	hunter-gatherers	folate rich-diet	Africa	*NA*	*NA*	Schlebusch *et al* (2012)
	Khomani2 (South Africa)	39	hunter-gatherers	folate rich-diet	Africa	*NA*	*NA*	Schlebusch *et al* (2012)
	Kung Vasekela	*NA*	hunter-gatherers	folate rich-diet	Africa	*NA*	*NA*	HGDP-CEPH
	Angola	29	agriculturalist	folate poor-diet	Africa	*NA*	*NA*	*this study*

c. (*NAT2*6*)	Mozambique	30	agriculturalist	folate poor-diet	Africa	*NA*	*NA*	*this study*
	Equatorial Guinea	87	agriculturalist	folate poor-diet	Africa	*NA*	*NA*	*this study*
	N. Bantu	12	agriculturalist	folate poor-diet	Africa	*NA*	*NA*	HGDP-CEPH
	Mandenka	24	agriculturalist	folate poor-diet	Africa	*NA*	*NA*	HGDP-CEPH
	S. Bantu	8	agriculturalist	folate poor-diet	Africa	*NA*	*NA*	HGDP-CEPH
	Luhya	88	agriculturalist	folate poor-diet	Africa	*NA*	*NA*	HapMap Ph3
	Mozabite	30	agriculturalist	folate poor-diet	Africa	*NA*	*NA*	HGDP-CEPH
	Yoruba	25	agriculturalist	folate poor-diet	Africa	*NA*	*NA*	HGDP-CEPH
	Portugal	47	agriculturalist	folate poor-diet	Europe	*NA*	*NA*	*this study*
	Basque	24	agriculturalist	folate poor-diet	Europe	*NA*	*NA*	HGDP-CEPH
	Bergamo	14	agriculturalist	folate poor-diet	Europe	*NA*	*NA*	HGDP-CEPH
	French	29	agriculturalist	folate poor-diet	Europe	*NA*	*NA*	HGDP-CEPH
	Orcadian	16	agriculturalist	folate poor-diet	Europe	*NA*	*NA*	HGDP-CEPH
	Russian	25	agriculturalist	folate poor-diet	Europe	*NA*	*NA*	HGDP-CEPH
	Sardinian	28	agriculturalist	folate poor-diet	Europe	*NA*	*NA*	HGDP-CEPH
	Tuscan1	8	agriculturalist	folate poor-diet	Europe	*NA*	*NA*	HGDP-CEPH
	Tuscan2	89	agriculturalist	folate poor-diet	Europe	*NA*	*NA*	HapMap Ph3
	Adygei	17	agriculturalist	folate poor-diet	Middle Eastern	*NA*	*NA*	HGDP-CEPH
	Druze	48	agriculturalist	folate poor-diet	Middle Eastern	*NA*	*NA*	HGDP-CEPH
	Palestinian	51	agriculturalist	folate poor-diet	Middle Eastern	*NA*	*NA*	HGDP-CEPH
	Burusho	25	agriculturalist	folate poor-diet	South Asia	*NA*	*NA*	HGDP-CEPH
	Kalash	25	agriculturalist	folate poor-diet	South Asia	*NA*	*NA*	HGDP-CEPH
	Pathan	25	agriculturalist	folate poor-diet	South Asia	*NA*	*NA*	HGDP-CEPH
	Sindhi	25	agriculturalist	folate poor-diet	South Asia	*NA*	*NA*	HGDP-CEPH
	Xibo	9	agriculturalist	folate poor-diet	South Asia	*NA*	*NA*	HGDP-CEPH
	Gujarati	88	agriculturalist	folate poor-diet	South Asia	*NA*	*NA*	HapMap Ph3
	Cambodian	11	agriculturalist	folate poor-diet	East Asia	*NA*	*NA*	HGDP-CEPH
	Dai	10	agriculturalist	folate poor-diet	East Asia	*NA*	*NA*	HGDP-CEPH
	Han	45	agriculturalist	folate poor-diet	East Asia	*NA*	*NA*	HGDP-CEPH
	Japanese	31	agriculturalist	folate poor-diet	East Asia	*NA*	*NA*	HGDP-CEPH
	Lahu	10	agriculturalist	folate poor-diet	East Asia	*NA*	*NA*	HGDP-CEPH
	Miaozu	10	agriculturalist	folate poor-diet	East Asia	*NA*	*NA*	HGDP-CEPH
	Naxi	10	agriculturalist	folate poor-diet	East Asia	*NA*	*NA*	HGDP-CEPH
	She	10	agriculturalist	folate poor-diet	East Asia	*NA*	*NA*	HGDP-CEPH
	Tujia	10	agriculturalist	folate poor-diet	East Asia	*NA*	*NA*	HGDP-CEPH
	Yizu	10	agriculturalist	folate poor-diet	East Asia	*NA*	*NA*	HGDP-CEPH
	Daur	10	agriculturalist	folate poor-diet	East Asia	*NA*	*NA*	HGDP-CEPH
	Afar (Ethiopia)	12	agriculturalist	folate poor-diet	Africa	*NA*	*NA*	Pagani *et al* (2012)
	Amhara1	*NA*	agriculturalist	folate poor-diet	Africa	*NA*	*NA*	HapMap Ph3
	Amhara2 (Ethiopia)	26	agriculturalist	folate poor-diet	Africa	*NA*	*NA*	Pagani *et al* (2012)
	Anuak (Ethiopia)	23	agriculturalist	folate poor-diet	Africa	*NA*	*NA*	Pagani *et al* (2012)
	Ari Blacksmith (Ethiopia)	17	agriculturalist	folate poor-diet	Africa	*NA*	*NA*	Pagani *et al* (2012)
	Ari cultivator (Ethiopia)	24	agriculturalist	folate poor-diet	Africa	*NA*	*NA*	Pagani *et al* (2012)
	Tigray (Ethiopia)	21	agriculturalist	folate poor-diet	Africa	*NA*	*NA*	Pagani *et al* (2012)
	Wolayta (Ethiopia)	8	agriculturalist	folate poor-diet	Africa	*NA*	*NA*	Pagani *et al* (2012)
	SEBantu (Herero_Namibia)	12	agriculturalist	folate poor-diet	Africa	*NA*	*NA*	Schlebusch *et al* (2012)
	SWBantu (South Africa)	20	agriculturalist	folate poor-diet	Africa	*NA*	*NA*	Schlebusch *et al* (2012)
	Uganda	110	pastoralist	folate poor-diet	Africa	*NA*	*NA*	*this study*
	Masaai	143	pastoralist	folate poor-diet	Africa	*NA*	*NA*	HapMap Ph3
	Bedouin	49	pastoralist	folate poor-diet	South Asia	*NA*	*NA*	HGDP-CEPH
	Brahui	25	pastoralist	folate poor-diet	South Asia	*NA*	*NA*	HGDP-CEPH
	Hazara	25	pastoralist	folate poor-diet	South Asia	*NA*	*NA*	HGDP-CEPH
	Makrani	25	pastoralist	folate poor-diet	South Asia	*NA*	*NA*	HGDP-CEPH
	Uygur	10	pastoralist	folate poor-diet	South Asia	*NA*	*NA*	HGDP-CEPH
	Tu	10	pastoralist	folate poor-diet	South Asia	*NA*	*NA*	HGDP-CEPH
	Mongola	10	pastoralist	folate poor-diet	East Asia	*NA*	*NA*	HGDP-CEPH
	Balochi	25	pastoralist	folate poor-diet	East Asia	*NA*	*NA*	HGDP-CEPH
	Yakut	25	pastoralist	folate poor-diet	East Asia	*NA*	*NA*	HGDP-CEPH
	Somali (Ethiopia)	17	pastoralist	folate poor-diet	Africa	*NA*	*NA*	Pagani *et al* (2012)
	Gumuz (Ethiopia)	19	pastoralist	folate poor-diet	Africa	*NA*	*NA*	Pagani *et al* (2012)
	Nama (Namibia)	20	pastoralist	folate poor-diet	Africa	*NA*	*NA*	Schlebusch *et al* (2012)
	Uganda	110	pastoralist	folate poor-diet	Africa	*NA*	*NA*	*this study*
	Masaai	143	pastoralist	folate poor-diet	Africa	*NA*	*NA*	HapMap Ph3
	Oroqen	10	hunter-gatherer	folate rich-diet	East Asia	*NA*	*NA*	HGDP-CEPH
	Hezhen	10	hunter-gatherer	folate rich-diet	East Asia	*NA*	*NA*	HGDP-CEPH
	Maritime Chukchee	*NA*	hunter-gatherer	folate rich-diet	East Asia	*NA*	*NA*	HGDP-CEPH
	Naukan Yup'ik	*NA*	hunter-gatherer	folate rich-diet	East Asia	*NA*	*NA*	HGDP-CEPH
	Khoisan Namibia	62	hunter-gatherer	folate rich-diet	Africa	*NA*	*NA*	*this study*

	San	7	hunter-gatherer	folate rich-diet	Africa	*NA*	*NA*	HGDP-CEPH
	Baka Pygmies	39	hunter-gatherer	folate rich-diet	Africa	*NA*	*NA*	*this study*
	Biaka Pygmy	36	hunter-gatherer	folate rich-diet	Africa	*NA*	*NA*	HGDP-CEPH
	Mbuti Pygmy	15	hunter-gatherer	folate rich-diet	Africa	*NA*	*NA*	HGDP-CEPH
	Khomani1 (South Africa)	35	hunter-gatherers	folate rich-diet	Africa	*NA*	*NA*	Henn *et al* (2011)
	Hadza (Tanzania)	20	hunter-gatherers	folate rich-diet	Africa	*NA*	*NA*	Henn *et al* (2011)
	Sandawe (Tanzania)	35	hunter-gatherers	folate rich-diet	Africa	*NA*	*NA*	Henn *et al* (2011)
	Khwe (Angola)	17	hunter-gatherers	folate rich-diet	Africa	*NA*	*NA*	Schlebusch *et al* (2012)
	!Xun (Angola)	19	hunter-gatherers	folate rich-diet	Africa	*NA*	*NA*	Schlebusch *et al* (2012)
	Gui and Ghana (Botswana)	15	hunter-gatherers	folate rich-diet	Africa	*NA*	*NA*	Schlebusch *et al* (2012)
	Ju/'hoansi (Namibia)	18	hunter-gatherers	folate rich-diet	Africa	*NA*	*NA*	Schlebusch *et al* (2012)
	Karretjie (South Africa)	20	hunter-gatherers	folate rich-diet	Africa	*NA*	*NA*	Schlebusch *et al* (2012)
	Khomani2 (South Africa)	39	hunter-gatherers	folate rich-diet	Africa	*NA*	*NA*	Schlebusch *et al* (2012)
	Kung Vasekela	*NA*	hunter-gatherers	folate rich-diet	Africa	*NA*	*NA*	HGDP-CEPH
c. (CYP3A5)	Angola	29	agriculturalist	salt rich-diet	Africa	15S-0	*NA*	*this study*
	Mozambique	30	agriculturalist	salt rich-diet	Africa	30-15S	*NA*	*this study*
	Equatorial Guinea	87	agriculturalist	salt rich-diet	Africa	0-15N	*NA*	*this study*
	N. Bantu	12	agriculturalist	salt rich-diet	Africa	15S-0	*NA*	HGDP-CEPH
	Mandenka	24	agriculturalist	salt rich-diet	Africa	0-15N	*NA*	HGDP-CEPH
	S. Bantu	8	agriculturalist	salt rich-diet	Africa	30-15S	*NA*	HGDP-CEPH
	Luhya	88	agriculturalist	salt rich-diet	Africa	0-15N	*NA*	HapMap Ph3
	Mozabite	30	agriculturalist	salt rich-diet	Africa	30-45N	*NA*	HGDP-CEPH
	Yoruba	25	agriculturalist	salt rich-diet	Africa	0-15N	*NA*	HGDP-CEPH
	Portugal	47	agriculturalist	salt rich-diet	Europe	30-45N	*NA*	*this study*
	Basque	24	agriculturalist	salt rich-diet	Europe	30-45N	*NA*	HGDP-CEPH
	Bergamo	14	agriculturalist	salt rich-diet	Europe	>45N	*NA*	HGDP-CEPH
	French	29	agriculturalist	salt rich-diet	Europe	>45N	*NA*	HGDP-CEPH
	Orcadian	16	agriculturalist	salt rich-diet	Europe	>45N	*NA*	HGDP-CEPH
	Russian	25	agriculturalist	salt rich-diet	Europe	30-45N	*NA*	HGDP-CEPH
	Sardinian	28	agriculturalist	salt rich-diet	Europe	30-45N	*NA*	HGDP-CEPH
	Tuscan1	8	agriculturalist	salt rich-diet	Europe	30-45N	*NA*	HGDP-CEPH
	Tuscan2	89	agriculturalist	salt rich-diet	Europe	30-45N	*NA*	HapMap Ph3
	Adygei	17	agriculturalist	salt rich-diet	Middle Eastern	30-45	*NA*	HGDP-CEPH
	Druze	48	agriculturalist	salt rich-diet	Middle Eastern	30-45N	*NA*	HGDP-CEPH
	Palestinian	51	agriculturalist	salt rich-diet	Middle Eastern	30-45N	*NA*	HGDP-CEPH
	Burusho	25	agriculturalist	salt rich-diet	South Asia	30-45N	*NA*	HGDP-CEPH
	Kalash	25	agriculturalist	salt rich-diet	South Asia	30-45N	*NA*	HGDP-CEPH
	Pathan	25	agriculturalist	salt rich-diet	South Asia	30-45N	*NA*	HGDP-CEPH
	Sindhi	25	agriculturalist	salt rich-diet	South Asia	15-30N	*NA*	HGDP-CEPH
	Xibo	9	agriculturalist	salt rich-diet	South Asia	30-45N	*NA*	HGDP-CEPH
	Gujarati	88	agriculturalist	salt rich-diet	South Asia	15-30N	*NA*	HapMap Ph3
	Cambodian	11	agriculturalist	salt rich-diet	East Asia	0-15N	*NA*	HGDP-CEPH
	Dai	10	agriculturalist	salt rich-diet	East Asia	15-30N	*NA*	HGDP-CEPH
	Han	45	agriculturalist	salt rich-diet	East Asia	30-45N	*NA*	HGDP-CEPH
	Japanese	31	agriculturalist	salt rich-diet	East Asia	30-45N	*NA*	HGDP-CEPH
	Lahu	10	agriculturalist	salt rich-diet	East Asia	15-30N	*NA*	HGDP-CEPH
	Miaozu	10	agriculturalist	salt rich-diet	East Asia	15-30N	*NA*	HGDP-CEPH
	Naxi	10	agriculturalist	salt rich-diet	East Asia	15-30N	*NA*	HGDP-CEPH
	She	10	agriculturalist	salt rich-diet	East Asia	15-30N	*NA*	HGDP-CEPH
	Tujia	10	agriculturalist	salt rich-diet	East Asia	15-30N	*NA*	HGDP-CEPH
	Yizu	10	agriculturalist	salt rich-diet	East Asia	15-30N	*NA*	HGDP-CEPH
	Daur	10	agriculturalist	salt rich-diet	East Asia	30-45N	*NA*	HGDP-CEPH
	Afar (Ethiopia)	12	agriculturalist	salt rich-diet	Africa	0-15N	*NA*	Pagani *et al* (2012)
	Amhara2 (Ethiopia)	26	agriculturalist	salt rich-diet	Africa	0-15N	*NA*	Pagani *et al* (2012)
	Anuak (Ethiopia)	23	agriculturalist	salt rich-diet	Africa	0-15N	*NA*	Pagani *et al* (2012)
	Ari Blacksmith (Ethiopia)	17	agriculturalist	salt rich-diet	Africa	0-15N	*NA*	Pagani *et al* (2012)
	Ari cultivator (Ethiopia)	24	agriculturalist	salt rich-diet	Africa	0-15N	*NA*	Pagani *et al* (2012)
	Tigray (Ethiopia)	21	agriculturalist	salt rich-diet	Africa	0-15N	*NA*	Pagani *et al* (2012)
	Wolayta (Ethiopia)	8	agriculturalist	salt rich-diet	Africa	0-15N	*NA*	Pagani *et al* (2012)
	SEBantu (Herero_Namibia)	12	agriculturalist	salt rich-diet	Africa	30-15S	*NA*	Schlebusch *et al* (2012)
	SWBantu (South Africa)	20	agriculturalist	salt rich-diet	Africa	30-15S	*NA*	Schlebusch *et al* (2012)
	Ghana	8	agriculturalist	salt rich-diet	Africa	0-15N	*NA*	Bryc *et al* (2010)
	Cameroon (Fang)	66	agriculturalist	salt rich-diet	Africa	0-15N	*NA*	Bryc *et al* (2010)
	C.A.R. (Kaba)	16	agriculturalist	salt rich-diet	Africa	0-15N	*NA*	Bryc *et al* (2010)
	Chad (Bulala)	15	agriculturalist	salt rich-diet	Africa	0-15N	*NA*	Bryc *et al* (2010)
	Congo	9	agriculturalist	salt rich-diet	Africa	0-15N	*NA*	Bryc *et al* (2010)
	Nigeria (Hausa)	12	agriculturalist	salt rich-diet	Africa	0-15N	*NA*	Bryc *et al* (2010)
	Uganda	110	pastoralist	salt rich-diet	Africa	0-15N	*NA*	*this study*

Masaai	143	pastoralist	salt rich-diet	Africa	15S-0	*NA*	HapMap Ph3
Bedouin	49	pastoralist	salt rich-diet	South Asia	30-45N	*NA*	HGDP-CEPH
Brahui	25	pastoralist	salt rich-diet	South Asia	30-45N	*NA*	HGDP-CEPH
Hazara	25	pastoralist	salt rich-diet	South Asia	30-45N	*NA*	HGDP-CEPH
Makrani	25	pastoralist	salt rich-diet	South Asia	15-30N	*NA*	HGDP-CEPH
Uygur	10	pastoralist	salt rich-diet	South Asia	30-45N	*NA*	HGDP-CEPH
Tu	10	pastoralist	salt rich-diet	South Asia	30-45N	*NA*	HGDP-CEPH
Mongola	10	pastoralist	salt rich-diet	East Asia	30-45N	*NA*	HGDP-CEPH
Balochi	25	pastoralist	salt rich-diet	East Asia	30-45N	*NA*	HGDP-CEPH
Yakut	25	pastoralist	salt rich-diet	East Asia	>45N	*NA*	HGDP-CEPH
Somali (Ethiopia)	17	pastoralist	salt rich-diet	Africa	0-15N	*NA*	Pagani *et al* (2012)
Gumuz (Ethiopia)	19	pastoralist	salt rich-diet	Africa	0-15N	*NA*	Pagani *et al* (2012)
Nama (Namibia)	20	pastoralist	salt rich-diet	Africa	30-15S	*NA*	Schlebusch *et al* (2012)
Khoisan Namibia	62	hunter-gatherer	salt poor-diet	Africa	30-15S	*NA*	*this study*
San	7	hunter-gatherer	salt poor-diet	Africa	30-15S	*NA*	HGDP-CEPH
Baka Pygmies	39	hunter-gatherer	salt poor-diet	Africa	0-15N	*NA*	*this study*
Biaka Pygmy	36	hunter-gatherer	salt poor-diet	Africa	0-15N	*NA*	HGDP-CEPH
Mbuti Pygmy	15	hunter-gatherer	salt poor-diet	Africa	0-15N	*NA*	HGDP-CEPH
Oroqen	10	hunter-gatherer	salt poor-diet	East Asia	>45N	*NA*	HGDP-CEPH
Hezhen	10	hunter-gatherer	salt poor-diet	East Asia	>45N	*NA*	HGDP-CEPH
Khomani1 (South Africa)	35	hunter-gatherers	salt poor-diet	Africa	30-15S	*NA*	Henn *et al* (2011)
Hadza (Tanzania)	20	hunter-gatherers	salt poor-diet	Africa	15S-0	*NA*	Henn *et al* (2011)
Sandawe (Tanzania)	35	hunter-gatherers	salt poor-diet	Africa	15S-0	*NA*	Henn *et al* (2011)
Khwe (Angola)	17	hunter-gatherers	salt poor-diet	Africa	30-15S	*NA*	Schlebusch *et al* (2012)
!Xun (Angola)	19	hunter-gatherers	salt poor-diet	Africa	15S-0	*NA*	Schlebusch *et al* (2012)
Gui and Ghana (Botswana)	15	hunter-gatherers	salt poor-diet	Africa	30-15S	*NA*	Schlebusch *et al* (2012)
Ju/’hoansi (Namibia)	18	hunter-gatherers	salt poor-diet	Africa	30-15S	*NA*	Schlebusch *et al* (2012)
Karretjie (South Africa)	20	hunter-gatherers	salt poor-diet	Africa	30-15S	*NA*	Schlebusch *et al* (2012)
Khomani2 (South Africa)	39	hunter-gatherers	salt poor-diet	Africa	30-15S	*NA*	Schlebusch *et al* (2012)
