# Supplementary material for: Exploring the relationship between lifestyles, diets and genetic adaptations in humans
Source: BMC Genet. 2015 May 28;16:55. doi: 10.1186/s12863-015-0212-1 (PMC4445807; doi:10.1186/s12863-015-0212-1)
Supplement: Additional file 3: Table S3. — Haplotype and phenotype frequencies for NAT2 gene. [file 12863_2015_212_MOESM3_ESM.pdf]

Table S3. Haplotype and phenotype frequencies for *NAT2* gene.

HAPLOTYPE	ANG	EQG	MOZ	UGN	BPY	KNA	PTG
**ACAG**	-	-	-	0.0056	-	-	-
**ACGG**	-	0.0091	0.0250	0.0064	-	-	-
**ATGG**	0.1053	0.0596	0.1250	0.0769	0.0135	-	-
**GCGG**	0.2894	0.3659	0.2250	0.3658	0.1622	0.0738	0.500
**GTAA**	-	0.0150	-	0.0111	-	0.0010	-
**GTAG**	0.3947	0.1975	0.300	0.300	0.2568	0.0482	0.1980
**GTGG**	0.2105	0.3117	0.300	0.2342	0.5676	0.7633	0.2604
**GCGA**	-	-	0.0250	-	-	-	-
**GTGA**	-	0.0350	-	-	-	0.1137	0.0417
**ATAA**	-	0.0063	-	-	-	-	-
**Slow**	0.5500	0.3735	0.4500	0.6750	0.1351	0.0164	0.5218
**Intermediate**	0.3000	0.5301	0.5000	0.2375	0.5135	0.3770	0.3478
**Fast**	0.1500	0.0964	0.0500	0.0875	0.3514	0.6066	0.1304

Populations' abbreviations as referred in material and methods section.
