# Supplementary figures and images for: Exploring the relationship between lifestyles, diets and genetic adaptations in humans
Source: BMC Genet. 2015 May 28;16:55. doi: 10.1186/s12863-015-0212-1 (PMC4445807; doi:10.1186/s12863-015-0212-1)

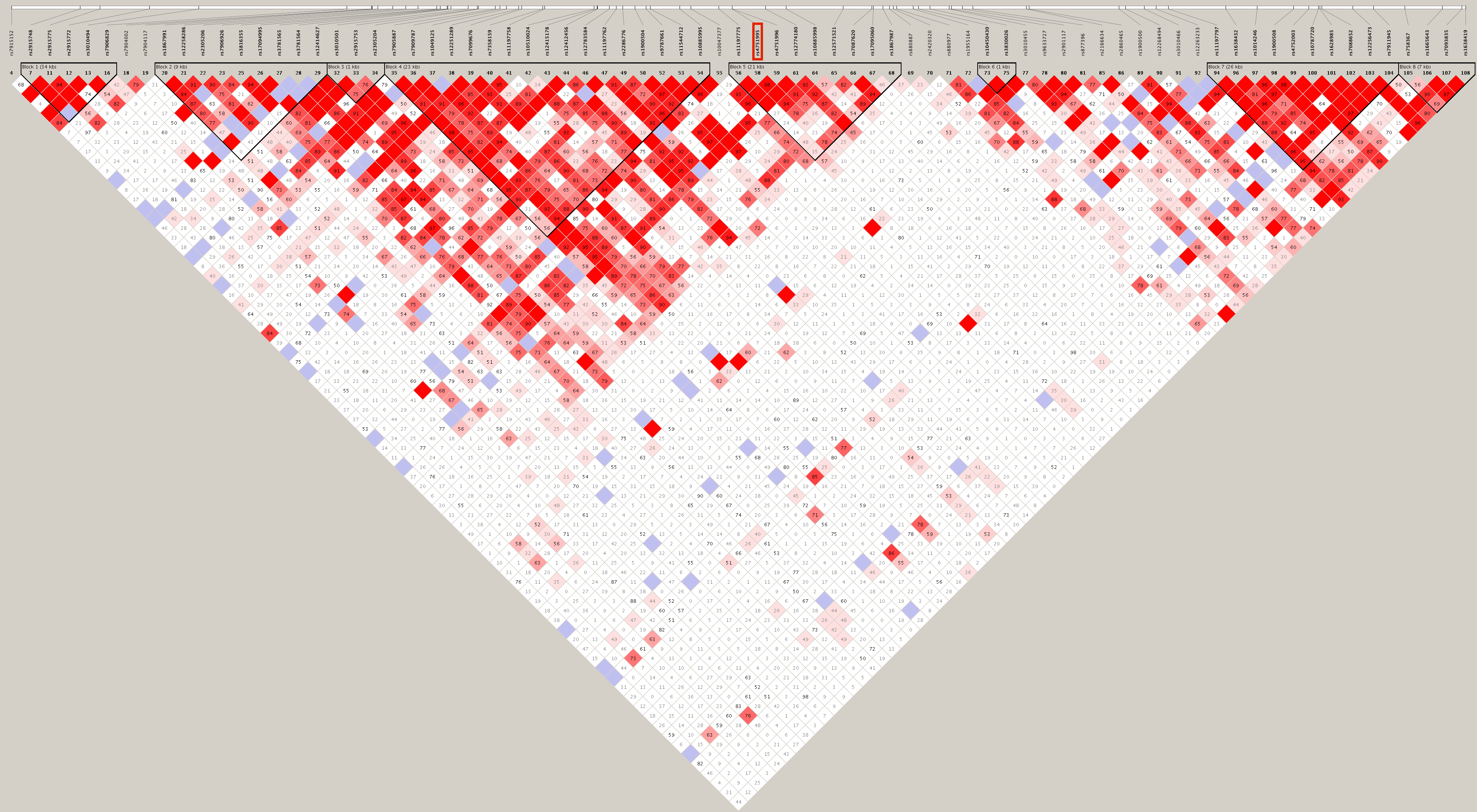

Supplement: Additional file 4: Figure S1. — LD patterns for PLRP2 in African populations from Pagani et al. (2012) [58]. The studied SNP is indicated by a red rectangle. In black triangles are represented the LD blocks. The degree of LD between pairs of markers is indicated by the |D’| statistic (|D’| = 1, red; |D’| < 1, shades of red). [file 12863_2015_212_MOESM4_ESM.png]

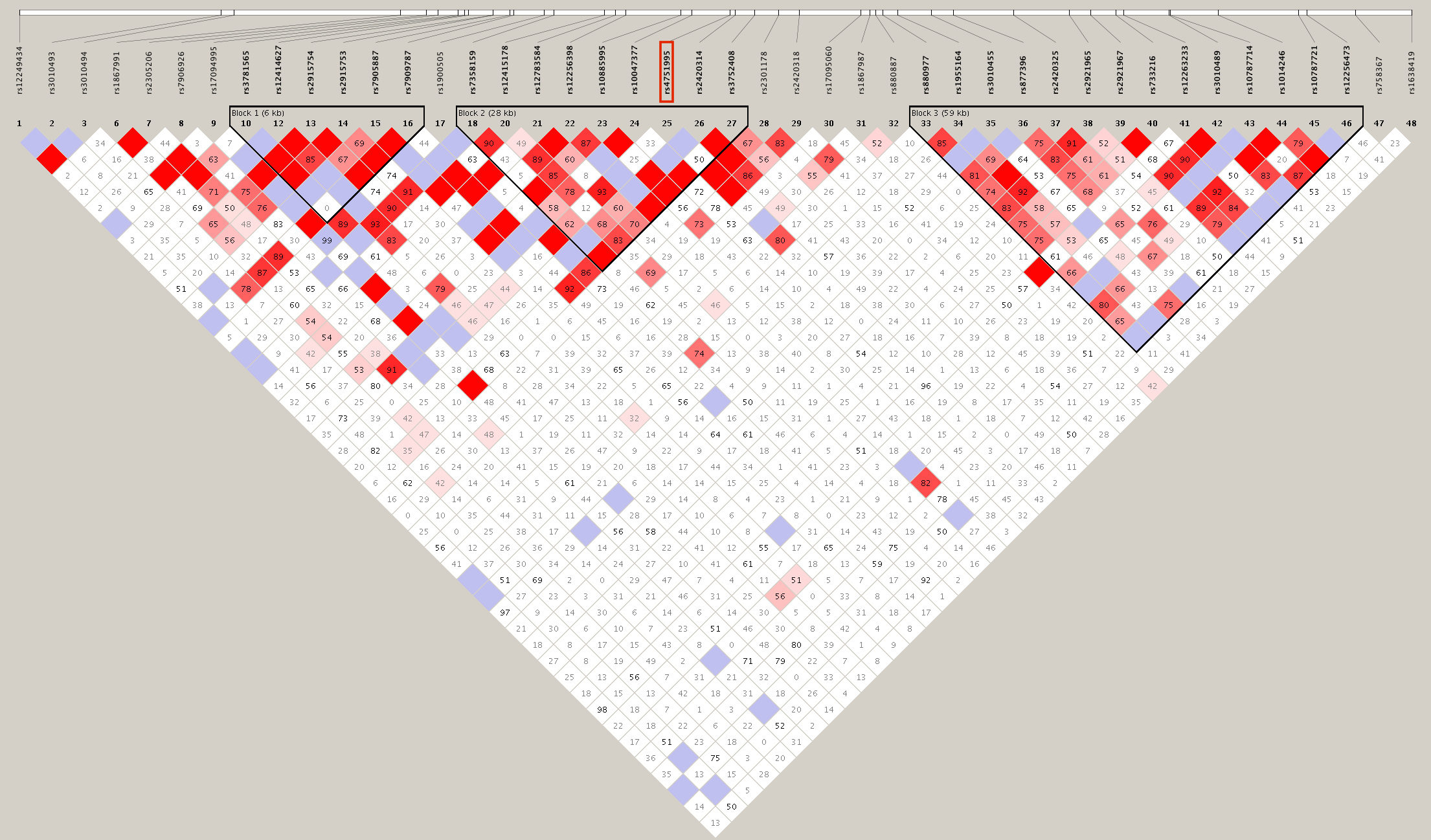

Supplement: Additional file 5: Figure S2. — LD patterns for PLRP2 in African populations from Henn et al. (2011) [57]. The studied SNP is indicated by a red rectangle. In black triangles are represented the LD blocks. The degree of LD between pairs of markers is indicated by the |D’| statistic (|D’| = 1, red; |D’| < 1, shades of red). [file 12863_2015_212_MOESM5_ESM.png]

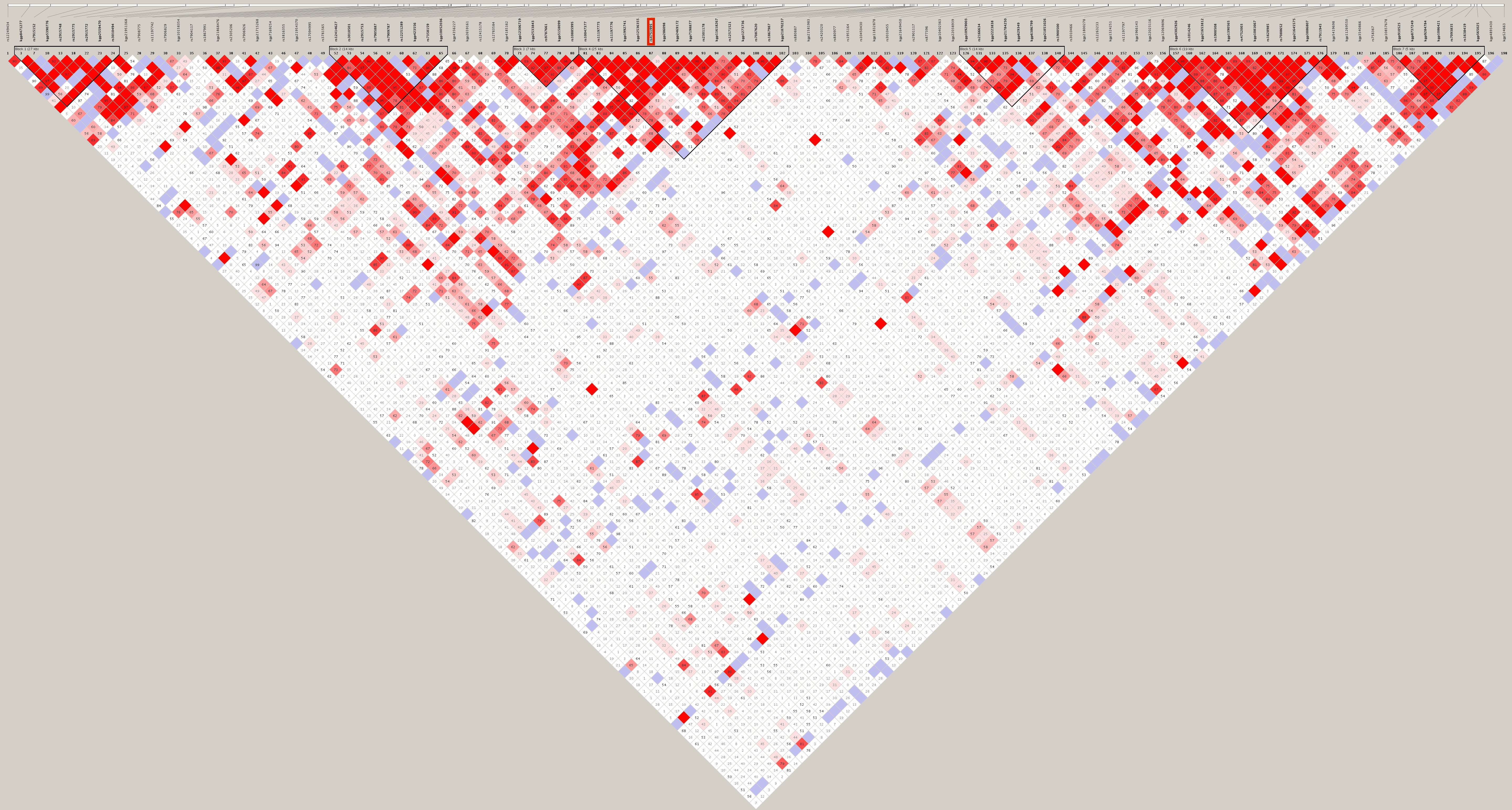

Supplement: Additional file 6: Figure S3. — LD patterns for PLRP2 in African populations from Schlebusch et al. (2012) [59]. The studied SNP is indicated by a red rectangle. In black triangles are represented the LD blocks. The degree of LD between pairs of markers is indicated by the |D’| statistic (|D’| = 1, red; |D’| < 1, shades of red). [file 12863_2015_212_MOESM6_ESM.png]

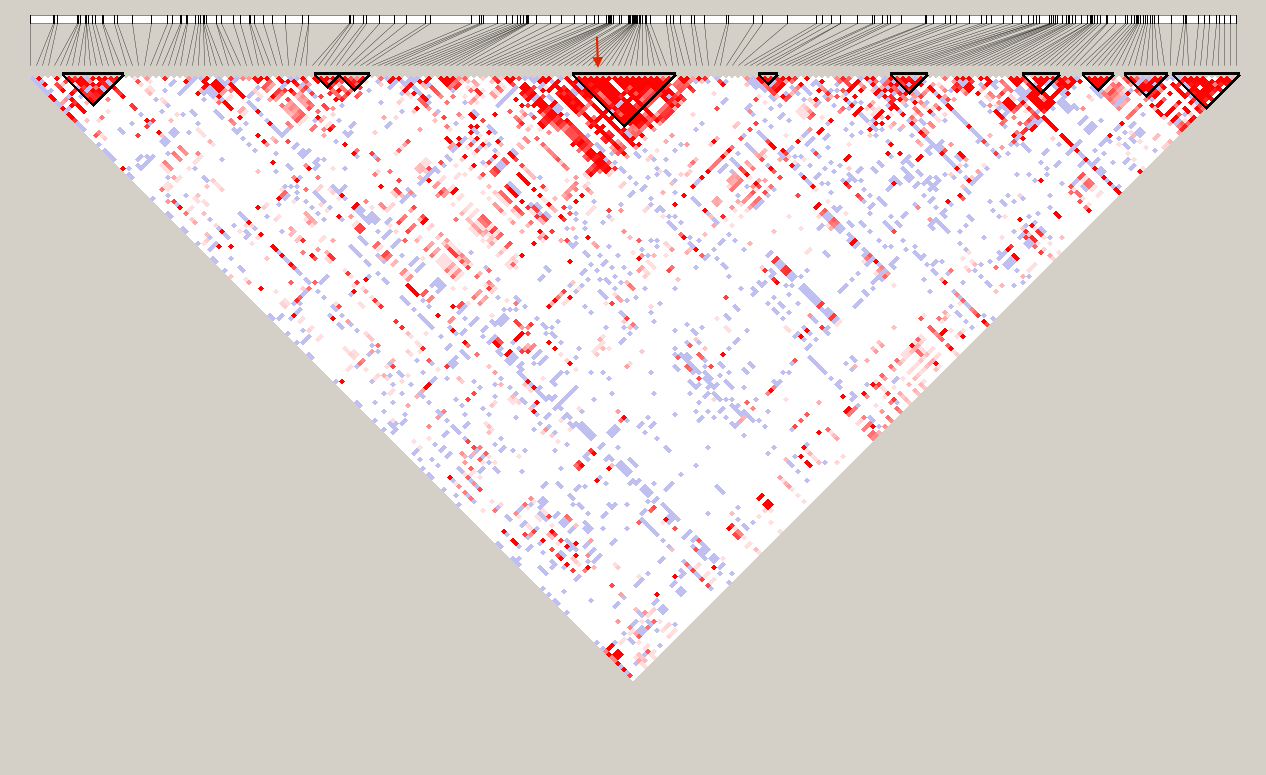

Supplement: Additional file 7: Figure S4. — LD patterns for NAT2 in African populations from Henn et al. (2011) [57]. A red arrow indicates the studied SNP. In black triangles are represented the LD blocks. The degree of LD between pairs of markers is indicated by the |D’| statistic (|D’| = 1, red; |D’| < 1, shades of red). [file 12863_2015_212_MOESM7_ESM.png]

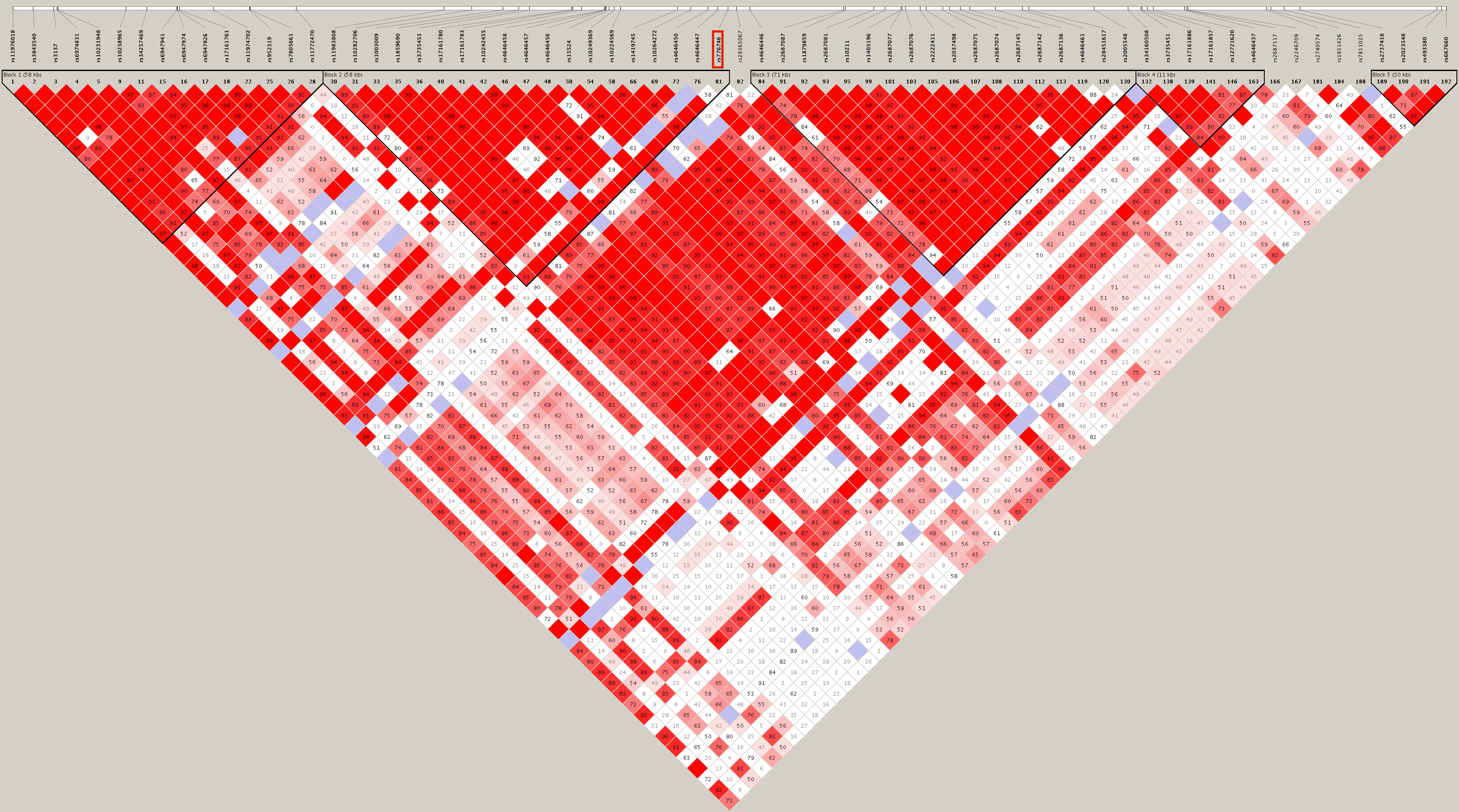

Supplement: Additional file 8: Figure S5. — LD patterns for CYP3A5 in African populations from Pagani et al. (2012) [58]. The studied SNP is indicated by a red rectangle. In black triangles are represented the LD blocks. The degree of LD between pairs of markers is indicated by the |D’| statistic (|D’| = 1, red; |D’| < 1, shades of red). [file 12863_2015_212_MOESM8_ESM.png]

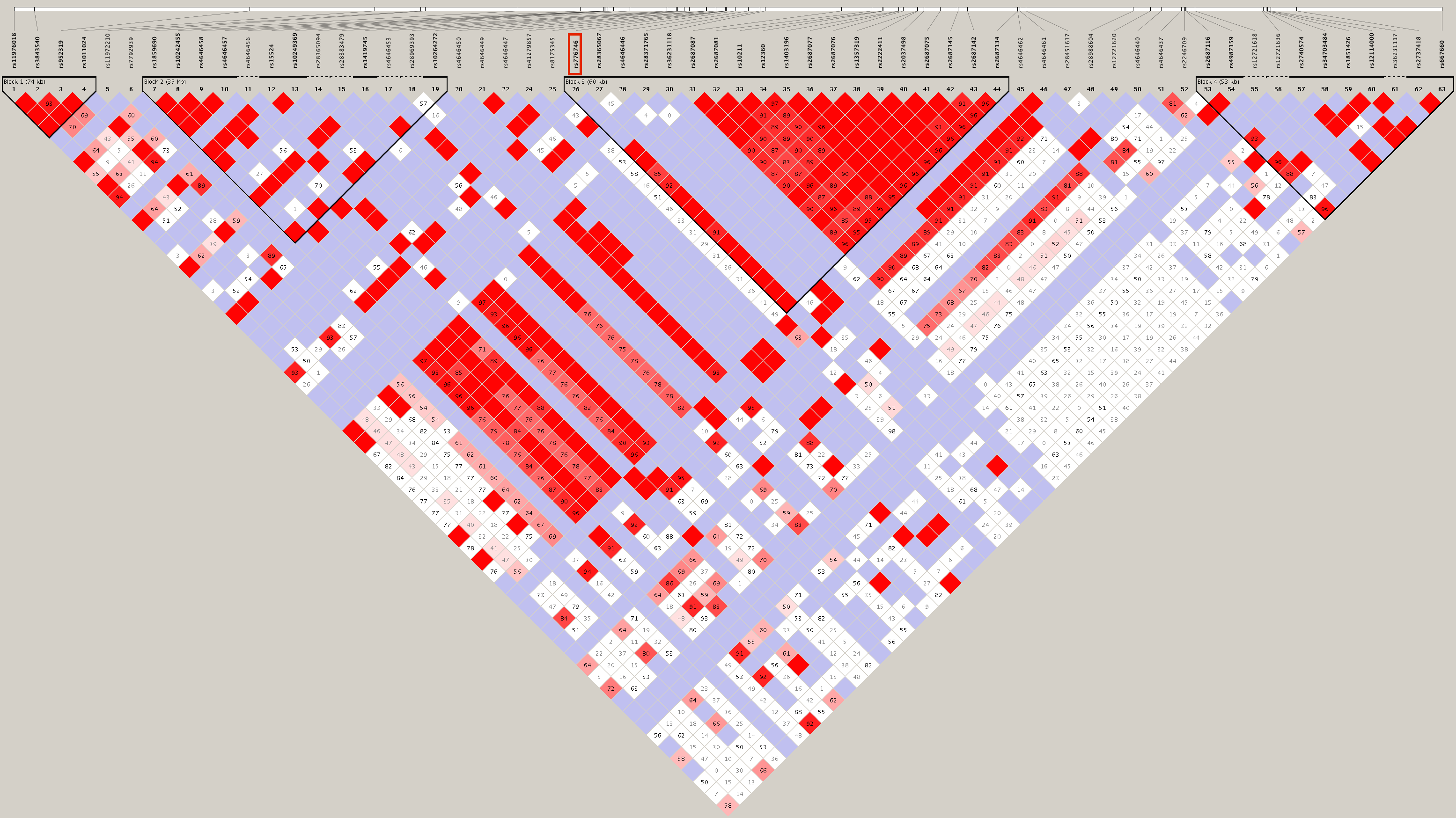

Supplement: Additional file 9: Figure S6. — LD patterns for CYP3A5 in African populations from Henn et al. (2011) [57]. The studied SNP is indicated by a red rectangle. In black triangles are represented the LD blocks. The degree of LD between pairs of markers is indicated by the |D’| statistic (|D’| = 1, red; |D’| < 1, shades of red). [file 12863_2015_212_MOESM9_ESM.png]

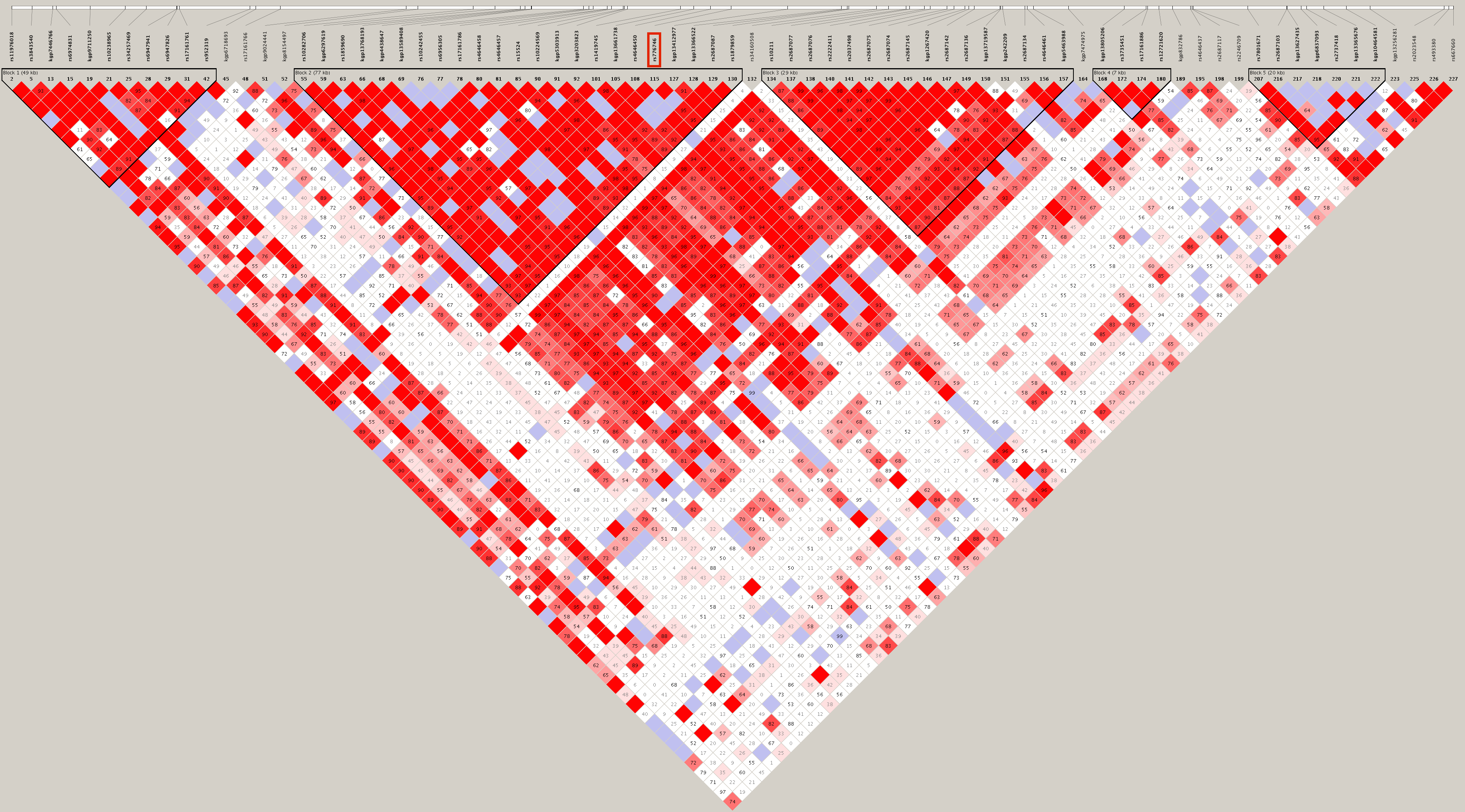

Supplement: Additional file 10: Figure S7. — LD patterns for CYP3A5 in African populations from Schlebusch et al. (2012) [59]. The studied SNP is indicated by a red rectangle. In black triangles are represented the LD blocks. The degree of LD between pairs of markers is indicated by the |D’| statistic (|D’| = 1, red; |D’| < 1, shades of red). [file 12863_2015_212_MOESM10_ESM.png]
