# Supplementary material for: Exploring the relationship between lifestyles, diets and genetic adaptations in humans
Source: BMC Genet. 2015 May 28;16:55. doi: 10.1186/s12863-015-0212-1 (PMC4445807; doi:10.1186/s12863-015-0212-1)
Supplement: Additional file 11: Table S4. — Amplification primers sequences. [file 12863_2015_212_MOESM11_ESM.pdf]

Table S4. Amplification primers sequences.

Gene	SNPs	Primer forward	Primer reverse	Size (bp)
AGXT	c.32C>T	TCCACCAATCCTCACCTCTC	CCCCACTCCTACCTGGTACA	288
PLRP2	c.1074G>A	CTGGAGGATTCAGAGCTTGG	CAAAGCAATCCTGATGTACCC	163
MTRR	c.1130A>G	CGTGATCTGCCCTAACAGTG	ACAGCATCAGGGCTGTTACC	135
NAT2	c.191G>A; c.341T>C; c.590G>A; c.857G>A	TAACATGCATTGTGGGCAAG	GAGTTGGGTGATACATACACAAGG	797
CYP3A5	c.219-237G>A	ACCACCCAGCTTAACGAATG	GGTCCAAACAGGGAAGAGATA	94
