# Supplementary material for: Exploring the relationship between lifestyles, diets and genetic adaptations in humans
Source: BMC Genet. 2015 May 28;16:55. doi: 10.1186/s12863-015-0212-1 (PMC4445807; doi:10.1186/s12863-015-0212-1)
Supplement: Additional file 12: Table S5. — Mini-sequencing primers sequences. [file 12863_2015_212_MOESM12_ESM.pdf]

Table S5. Mini-sequencing primers sequences.

Gene	SNP	Minisequencing primer seq 5'>3'	Size (bp)	Detection	Mutation	final size (bp)
AGXT	c.32C>T	GCTTGAGCAGGGCCTTG	17	A/G	C/T	18
PLRP2	c.1074G>A	ctgacaaGTATTTCTTTGGACAGGTTG	20	A/G	A/G	28
MTRR	c.1130A>G	tgactaaactaggtgccacgtcgtgaaagtctgacaaGAAAATAAAGGCAGACACAA	20	A/G	A/G	58
NAT2	c.191G>A	gtgaaagtctgacaaaactgactaaactaggtgccacgtcgtgaaagtctgacaaGACCTGGAGA**Y**ACCACCCACCC	22	T/C	A/G	78
	c.341T>C	ccacgtcgtgaaagtctgacaaCCTTCTCCTGCAGGTGACCA	20	T/C	T/C	43
	c.590G>A	tgacaaaactgactaaactaggtgccacgtcgtgaaagtctgacaaACTTATTTA**C**GCTTGAACCTC	21	A/G	A/G	68
	c.857G>A	ctaggtgccacgtcgtgaaagtctgacaaTGCCCA**M**ACCTGGTGATG	18	A/G	A/G	48
CYP3A5	c.219-237G>A	aagtctgacaaGGTCCAAACAGGGAAGAGATA	21	T/C	A/G	33

aactgactaaactaggtgccacgtcgtgaaagtctgacaa is the tale used by convention; in **bold** are highlighted the polymorphisms inside the sequencing primers.
